# Association between adverse childhood experiences and suicidal behavior in affective disorders: A systematic review and meta-analysis

**DOI:** 10.1192/j.eurpsy.2025.2452

**Published:** 2025-05-28

**Authors:** Valentina Baldini, Carolina Gottardi, Ramona Di Stefano, Lorenzo Vittorio Rindi, Gabriele Pazzocco, Giorgia Varallo, Marianna Purgato, Diana De Ronchi, Corrado Barbui, Giovanni Ostuzzi

**Affiliations:** 1 Department of Biomedical and Neuromotor Sciences, University of Bologna, Bologna, Italy; 2 Department of Biomedical, Metabolic and Neural Sciences, University of Modena and Reggio Emilia, Modena, Italy; 3 WHO Collaborating Centre for Research and Training in Mental Health and Service Evaluation, Department of Neuroscience, Biomedicine and Movement Sciences, Section of Psychiatry, Verona, Italy; 4 Department of Biotechnological and Applied Clinical Sciences, University of L’Aquila, L’Aquila, Italy; 5 Department of Systems Medicine, Tor Vergata University, Rome, Italy

**Keywords:** adverse childhood experiences, affective disorders, childhood trauma, suicide behaviors, suicide

## Abstract

**Background:**

Exposure to Adverse Childhood Experiences (ACEs) might increase the risk of suicide behaviors in the general adult population, while this association in individuals with affective disorders remains less characterized.

**Methods:**

A comprehensive search was conducted in MEDLINE, PsycINFO, CINAHL, Web of Science, Scopus, and PubMed up to July 10th, 2024. Observational studies that compared the risk of suicide behaviors in individuals exposed and unexposed to ACEs were included. Pairwise random-effects meta-analyses were conducted, and the certainty of evidence was assessed with validated criteria.

**Results:**

A total of 41 studies from 17 countries, comprising 19,588 participants, were analyzed. The main findings indicated a significant association between ACEs and suicidal behaviors, with an odds ratio (OR) of 1.98 (95% confidence interval [CI] 1.74–2.26), and a “highly suggestive” strength of association. This was consistent across diagnostic subgroups (i.e., Major Depressive Disorders, Bipolar Disorders, and mixed diagnoses). The association was confirmed for any ACE, with sexual abuse being the most frequently reported and showing the highest risk (OR 2.24; 95% CI 1.90–2.64), for suicidal ideation (OR 2.16; 95% CI 1.42–3.29), and for suicide attempts (OR 1.95; 95% CI 1.70–2.25), while death by suicide and non-suicidal self-injury were underreported. Meta-regression analyses did not suggest potential moderators, though underreporting was noted.

**Conclusions:**

This meta-analysis shows that exposure to ACEs nearly doubles the risk of suicide behaviors in individuals with affective disorders, warranting the targeted clinical, research, and policy measures to timely address this global mental health issue.

## Introduction

Adverse childhood experiences (ACEs), including abuse (physical, sexual, and emotional) and neglect (physical and emotional), are highly prevalent and represent public health concerns due to their deleterious impact on physical and mental well-being [1, 2]. The overall prevalence of lifetime childhood maltreatment has been estimated to approach 30% in population-based samples [3]. A large database study in the U.S. recently showed that up to 64% of adults reported at least one ACE, and up to 17% reported four or more ACEs [4]. The economic burden of ACEs in terms of health care, medical costs, and welfare is estimated to be around 124 billion in the U.S. alone [5].

Exposure to ACEs is associated with a higher risk of mental health disorders and suicidal behaviors in adulthood [6, 7], with a likely cumulative effect [8]. A meta-analysis of twin studies found a link between childhood sexual abuse and later suicide attempts, even after adjusting for genetic factors, family environment, and other risk factors [9]. Several theoretical frameworks help explain this association. Insecure attachment styles (e.g., disorganized or anxious-ambivalent) may promote emotional dysregulation [10, 11], while alexithymia and dysfunctional coping (e.g., avoidance, rumination, risk-taking) can further heighten suicide risk [12]. The interpersonal theory of suicide posits that individuals exposed to ACEs may be more prone to feelings of burdensomeness and thwarted belongingness [13]. Finally, chronic stress can alter key neurobiological processes, impairing emotional regulation through disruptions in threat detection, reward anticipation, and stress response [14].

Regarding specific sub-populations of individuals with mental health conditions, a clear association has been demonstrated between ACEs and an increased risk of suicidal behaviors in schizophrenia [15]. While meta-analytic evidence also indicates that childhood adversity adversely affects the long-term trajectory of affective disorders [16], our current understanding of how ACEs may influence suicidal behaviors in individuals with major depressive disorder and bipolar disorder remains fragmented. Several smaller-scale cross-sectional and longitudinal studies suggest that ACEs could exacerbate clinical characteristics, increase relapse rates, and heighten the risk of suicidality in both major depressive disorder and bipolar disorder [17, 18].

In particular, previous research has primarily focused on the general population or broader psychiatric samples, making it difficult to disentangle the specific risks faced by individuals with mood disorders. Different types of ACEs, such as physical, emotional, or sexual abuse, may also confer varying degrees of risk, yet relatively few studies have systematically compared these subtypes. Furthermore, small sample sizes and inconsistent outcome definitions (e.g., ideation versus attempts) have prevented firm conclusions. Therefore, a comprehensive meta-analysis focusing exclusively on major depressive disorder (MDD) and bipolar disorder (BD) is warranted to clarify the magnitude of the association across different forms of abuse, investigate potential moderators, and guide more tailored suicide prevention efforts in these high-risk populations.

Hence, to bridge this research gap, we conducted a systematic review and meta-analysis to assess the risk of suicide behaviors in individuals with a diagnosis of affective disorder who experienced different types of ACEs compared to those who did not experience them.

## Methods

We conducted a systematic review and meta-analysis following Preferred Reporting Items for Systematic Reviews and Meta-Analysis (PRISMA) guidelines [19]. The protocol was registered in advance (PROSPERO; CDR42023450850).

### Search strategy and study selection

Studies were included if they were observational prospective or retrospective cohorts, case–control, or cross-sectional studies that specifically recruited individuals (age 16 years or older) with a DSM- or ICD-based diagnosis of a mood disorder (i.e., major depressive disorder, bipolar disorder, or a mixed affective disorder diagnosis) or a diagnosis based on validated rating scales for these conditions. We set the age cutoff at 16 to capture late-adolescent individuals who may already present with clinically significant affective disorders and suicidal behaviors. Although 18 is often considered the threshold for adulthood, many clinical settings regard 16-year-olds as capable of receiving a formal psychiatric diagnosis, and it is well documented that first episodes of depression or bipolar disorder can emerge before 18 years of age.

Each study also had to report data on suicidal behaviors and/or non-suicidal self-injury (NSSI) in participants exposed compared to those unexposed. We considered ACEs as traumatic experiences occurring during childhood, including various forms of abuse, neglect, bullying, physical and verbal violence, and sexual abuse [20], as reported by authors in each primary study through clinical interviews or as assessed with validated rating scales, such as the Child Trauma Questionnaire Short Form (CTQ-SF) [21].

A literature search was carried out through the electronic databases PubMed, PsycINFO, Web of Science, and CINHAL from inception to July 10, 2024, with no language restrictions (see eAppendix, Supplement 2 for the full search strategy). After excluding duplicates, two researchers (among VB, CG, RDS, and LVR) independently screened titles, abstracts, and full texts of identified records. In case of disagreements on inclusion or exclusion, a consensus was reached by involving a senior researcher (GO, CB).

### Outcomes

The primary outcome was the risk of any suicide behavior (including suicidal ideation, suicide attempt, and death by suicide) in exposed versus unexposed individuals. Secondary analyses included the risk of any suicidal behavior in individuals exposed to different types of ACEs, namely physical abuse, physical neglect, sexual abuse, emotional abuse, and emotional neglect; the risk of each specific type of suicidal behavior (namely suicidal ideation, suicide attempt, and death by suicide) and NSSI in exposed versus unexposed individuals; and the risk of suicidal behavior as a function of the CTQ-SF score.

### Data extraction and analysis

**Figure 1.**
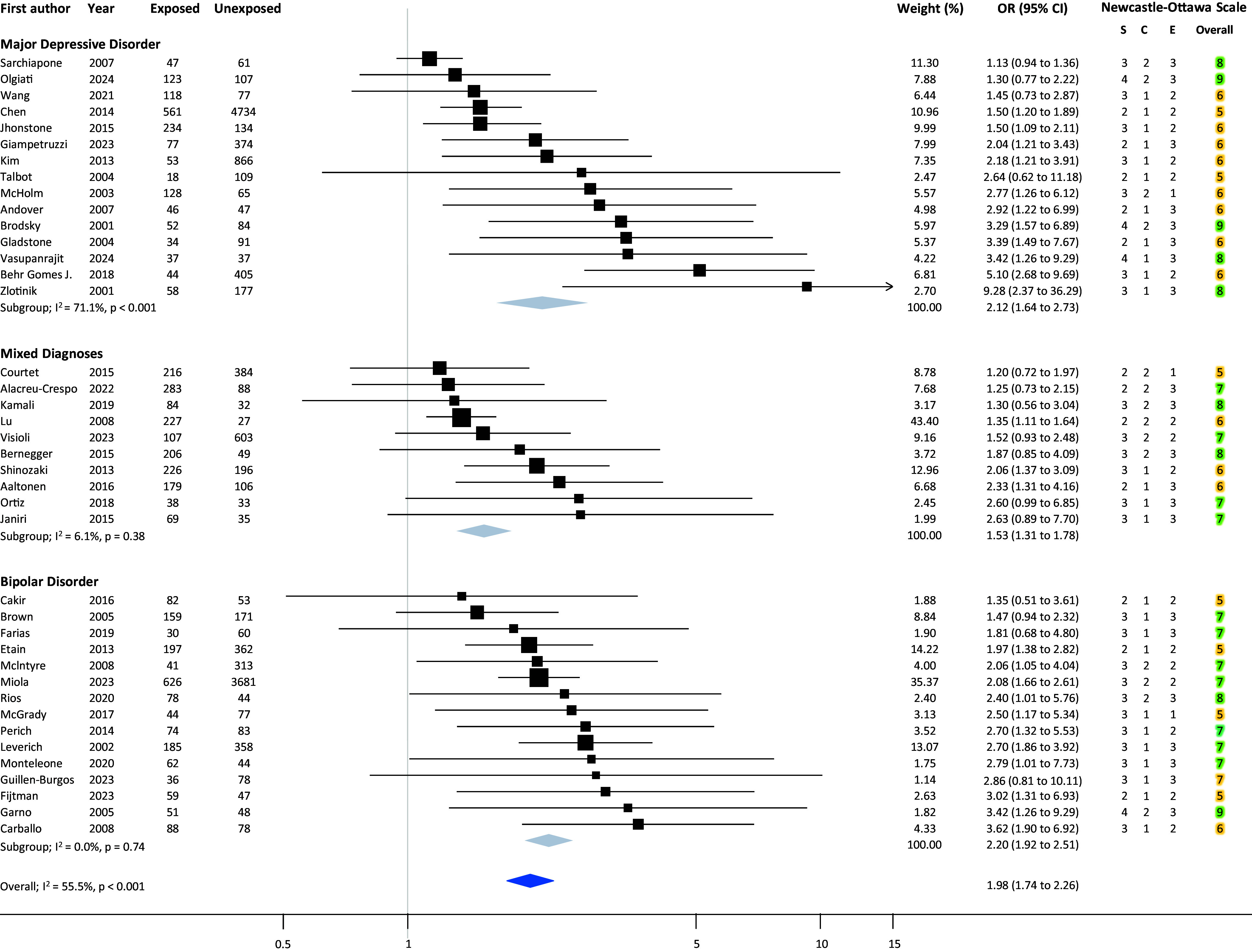
Forest plot showing the risk of any suicide behavior in individuals exposed to adverse childhood events compared to those unexposed, in the three diagnostic subgroups (i.e., major depressive disorder, mixed affective diagnoses, and bipolar disorders).

Statistical analyses were designed, performed, and reported following the Meta-analysis of Observational Studies in Epidemiology (MOOSE) [22]. When available, we extracted from primary studies the OR adjusted for sociodemographic and clinical characteristics. If this data was not available, we extracted from each study the number of individuals with and without the event of interest (i.e., suicide behavior) in the group of those exposed and unexposed to ACEs, and we calculated summary odds ratios (ORs) and 95% confidence intervals (CIs). If the number of individuals exposed and unexposed to ACEs was unavailable, we imputed it from mean scores and standard deviations of rating scales assessing ACEs, using cutoff scores indicating clinically relevant traumatic events, according to a validated methodology [23]. Data were pooled, and an overall estimate of ORs was obtained using the DerSimonian and Laird random-effects model, considering between-study variability [24]. We used the *I*-squared statistics (*I*^2^) and visual inspection of forest plots to investigate statistical heterogeneity. Unrestricted maximum likelihood random-effects meta-regression was employed to find whether sociodemographic and clinical variables mediated the association between ACE exposure and suicide behaviors in individuals with affective disorder (Figure 1).

### Critical appraisal assessment

Included studies were evaluated with the Newcastle-Ottawa Scale (NOS), which assesses the risk of bias in observational studies on three domains (selection, comparability, and exposure) and provides an overall score ranging from 1 (highest risk of bias) to 9 (lowest risk of bias) [25]. Two researchers (among CG, RDS, and LVR) assessed the risk independently, and disagreements were discussed with a third researcher (VB, GO). Furthermore, for the primary outcome, we evaluated the overall strength of the association according to the Umbrella Review Criteria [26] taking into account the following elements: (a) the number of cases (i.e., events) included in the analysis; (b) the magnitude of the *p*-value of the random-effect meta-analysis; (c) small study effects (an indicator of publication bias); (d) excess of significance bias; (e) predictive intervals; (f) nominal statistical significance of the largest study included; (g) between-study heterogeneity. According to this assessment, we classified the association as “Convincing” (Class I), “Highly Suggestive” (Class II), “Suggestive” (Class III), or “Weak” (Class IV). All statistical analyses were performed with Stata version 18.

### Subgroup and sensitivity analyses

For the primary outcome, we carried out a subgroup analysis separating different affective disorders (i.e., MDD, BD, and studies including mixed diagnoses). Further, we carried out the following sensitivity analyses: removing studies whose data were imputed from a continuous measure (i.e., rating scale score) and removing studies with a relevant risk of bias, defined as a total score of 5 or below on the Newcastle-Ottawa Scale. Moreover, we aimed to perform meta-regression analyses for the following variables possibly associated with suicidal risk [25], provided that at least 10 studies contributed: age, sex, marital status, having children, level of education, occupational level, being religious, physical illness, mental illness duration, age of onset, number of previous hospitalization, country income, tobacco smoking, alcohol, and substance abuse, family history of suicide behavior, any comorbid mental disorders.

## Results

**Figure 2.**
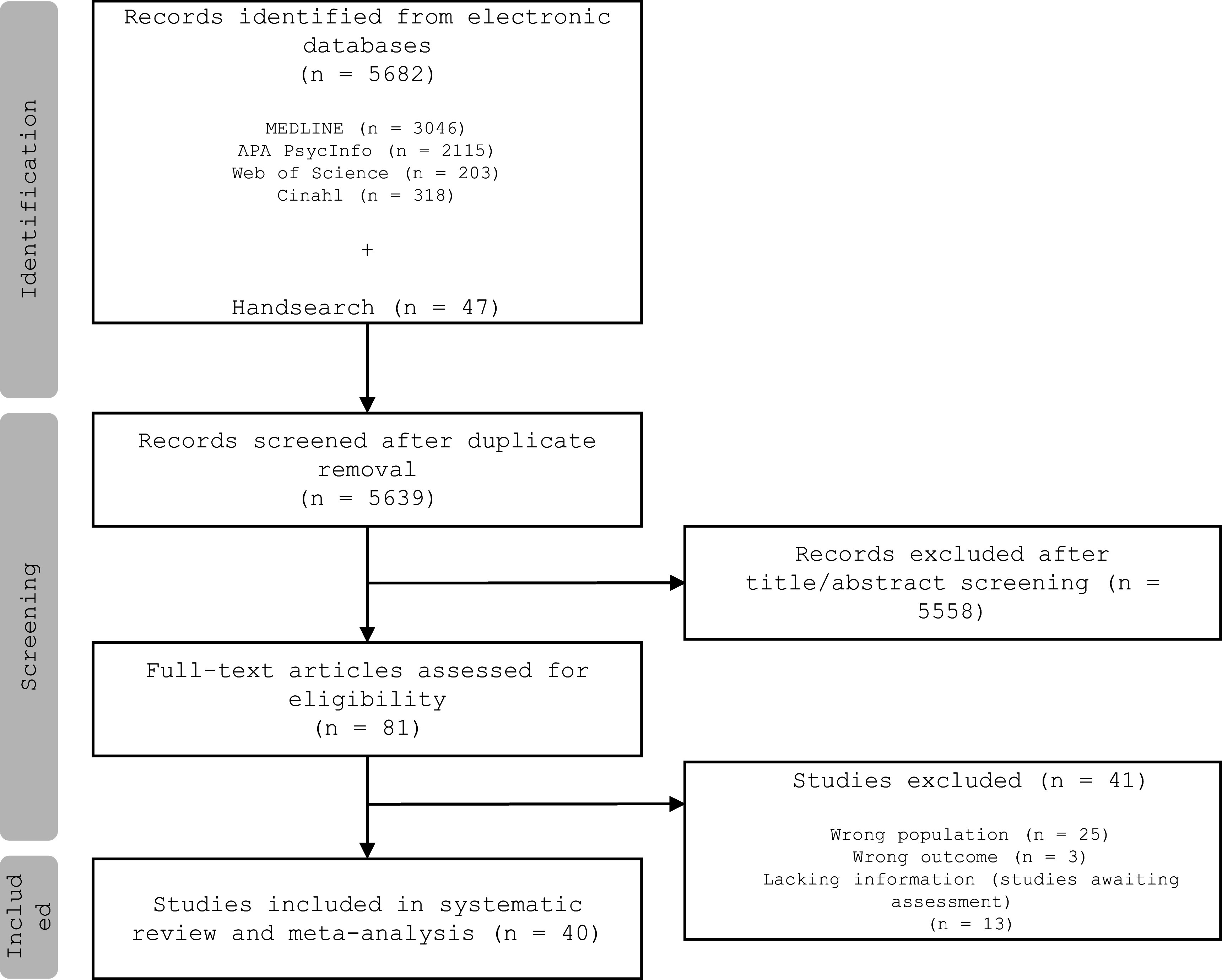
PRISMA flowchart showing the process of study selection.

A total of 5682 records were identified through a database search. We excluded 43 duplicates and screened 5639 titles and abstracts. A full-text review was carried out on 82 articles, with 41 studies meeting inclusion criteria and contributing to at least one meta-analysis (Figure 2).

Study characteristics are summarized in Table 1. In total, 41 studies were included, enrolling 19,588 participants from 17 different countries—predominantly in Europe (e.g., Italy, France, Finland), North and South America (e.g., USA, Brazil, Mexico), Asia (e.g., China, Korea), and Australia. the mean age of the participants was 37 years (standard deviation [SD] 9.8; median 40.6; inter-quartile range [IQR] 22.3–75.0), and the proportion of women was 66.2% (SD 18.9; median 65.0; IQR 6.4–100).

**Table 1.** Characteristics of included studies

First author, year	Country	Diagnoses in the SSD (criteria)	Mean age ± SD	Female participants (%)	Overall sample	Types of ACEs	Types of suicide behavior	NOS overall score
Aaltonen, 2016 [27]	Finland	BD (ICD–10/ DSM IV) 34.5%; MDD 65.5%	39.9 ± 13.0	72.8%	287	NS	SA; SI	6
Alacreu-Crespo, 2022 [28]	France	MDD (DSM IV)	42.07±0.65	69%	371	SexA, PA, PN, EA, EN	SA	7
Andover, 2007 [29]	USA	MDD (DSM III)	37.2±11.03	74%	93	PA, SexA	SA	6
Behr Gomes Jardim, 2018 [30]	Brazil	MDD (GDS)	75	64%	449	SexA, PA, PN, EA, EN	SA	6
Bernegger, 2015 [31]	Austria	MDD (DSM IV) 82.7%; BD 17.3%	NA	56.8%	255	SexA, PA, PN, EA, EN	SA; SI; NSSI	8
Brodsky, 2001 [32]	USA	MDD (DSM III)	36.5±11.91	63.9%	136	PA, SexA	SA	9
Brown, 2005 [33]	USA	BD I (DSM IV) 87.7%; BD II 12.3%	46.6±10	6.4%	330	PA, SexA	SA	7
Cakir, 2016 [34]	Turkey	BD I (DSM IV)	40.6±12.8	60.7%	135	SexA, PA, PN, EA, EN	SA	4
Carballo, 2008 [35]	USA	BD (DSM III)	35.9±10.75	69%	166	NA	SA	6
Chen, 2014 [36]	China	MDD (DSM IV)	44.4±8.9	100%	5,295	SexA	NA	5
Courtet, 2015 [37]	France	BD 30.5%; MDD 66.1% (DSM IV)	39.9±13.44	72%	600	SexA, PA, PN, EA, EN	SA	5
Etain, 2013 [38]	France, Norway	BD I 72.4%; BD II 21.5%; BD NOS 6.1% (DSM III)	40.6±13.6	60%	5,59	SexA, PA, PN, EA, EN	SA	5
Farias, 2019 [39]	Brazil	BD (MINI PLUS)	25.87±2.03	75%	90	NA	SA	7
Fijtman, 2023 [40]	USA	BD I; BD II (DSM IV)	35.02±8.62	32.1%	106	SexA; PA	SA	5
Garno, 2005 [41]	USA	BD I; BD II (DSM IV)	41.6±12.3	49%	99	NA	SA	9
Giampetruzzi, 2023 [42]	USA	MDD (DSM IV)	49.6±17.23	68%	451	SexA	SA	6
Gladstone, 2004 [43]	Australia	MDD (DSM IV)	38.4±12.3	100%	125	SexA	SA	6
Guillen‑Burgos, 2023 [44]	Colombia	BD I; BD II (DSM V)	31.5	40.6%	114	SexA, PA, PN, EA, EN	SA; SI	7
Janiri, 2015 [45]	Italy	BD I 55.8%; BD II 44.2% (DSM IV)	44.6±14.64	43%	104	SexA, PA, PN, EA, EN	SA	7
Jhonstone, 2015 [46]	New Zealand	MDD (DSM IV)	33.3±10.8	64%	368	SexA	SA	6
Kamali, 2019 [47]	USA	BD I (DIGS)	36.6±17.7	NA	116	NA	SA; SI	8
Kim, 2013 [48]	Korea	MDD (DSM IV)	46.1±15.9	73%	919	SexA; PA	SA	6
Leverich, 2002 [49]	USA	BD I 75%; BD II 22%; BD NOS 2% (DSM IV)	41±12	58%	631	SexA; PA	SA	7
Lu, 2008 [50]	USA	MDD 39%; BD 61% (DSM IV)	42.87±11	44%	254	SexA, PA	SA	6
McGrady, 2017 [51]	Spain	BD I BD II (DSM IV)	NA	69%	121	NA	SA	5
McHolm, 2003 [52]	USA	MDD (DSM III)	39.2	100%	193	PA	SA; SI	6
McIntyre, 2008 [53]	USA	BD (DSM IV)	37.74±11.25	55%	381	SexA; PA	SA	7
Miola, 2023 [54]	Italy	BD I 16.7%; BD II 16.4%; MDD 66.9% (DSM IV)	46.3	68%	4,307	SexA; PA	SA; SI	7
Monteleone, 2020 [55]	Italy	BD I; BD II (DSM IV)	48.4±12.32	53%	106	SexA, PA, PN, EA, EN	SA	7
Olgiati, 2024 [56]	Italy	MDD (DSM IV)	43.28±12.47	71%	230	NA	SI	9
Ortiz-Guzman, 2018 [57]	Mexico	MDD (DSM IV)	36.8±10.73	100%	71	SexA	NA	7
Perich, 2014 [58]	Australia	BD I; BD II (DSM IV)	NA	64%	157	SexA, PA	SA	7
Rios, 2020 [59]	Cile	BD (DSM IV)	45.5±13.9	65%	122	SexA, PA, PN, EA, EN	SA	8
Sarchiapone, 2007 [60]	Italy	MDD (DSM IV)	41.2±14.36	61%	108	SexA, PA, PN, EA, EN	SA	8
Shinozaki, 2013 [61]	USA	MDD; BD (DSM IV)	44.2±12.9	69.6%	422	NA	SA	6
Talbot, 2004 [62]	USA	MDD (DSM III)	68.8±11.4	100%	127	SexA	SA; SI	5
Vasupanrajit, 2024 [63]	Thailand	MDD (DSM V)	22.35	84%	74	SexA, PA, PN, EA, EN	SA; SI	8
Visioli, 2023 [64]	Italy	MDD 43.3%; BD 56.7% (DSM V)	49	62%	684	SexA, PA	NA	7
Wang, 2021 [65]	China	MDD (DSM V)	23	73%	197	SexA, PA, PN, EA, EN	SA; SI	6
Zlotinik, 2001 [66]	USA	MDD (DSM IV)	40.6±14.03	65%	235	SexA	SA	8

DIGS, Diagnostic Interview for Genetic Studies; SD, Suicide death; DSM, Diagnostic and Statistical Manual of Mental Disorders; EA, Emotional abuse; EN, Emotional neglect; ICD, International Classification of Diseases; NOS, Newcastle-Ottawa Scale; NS, Not Specified; PA, Physical abuse; PN, Physical neglect; SA, Suicide Attempt; SD, Standard Deviation; SexA, Sexual abuse; SI, Suicidal Ideation; BD, Bipolar Disorder; BD I, Bipolar Disorder Type 1; BD II, Bipolar Disorder Type II; BD NOS, Bipolar Disorder Not Otherwise Specified; Major Depressive Disorder: MDD; MINI-PLUS, Mini International Neuropsychiatric Interview-PLUS; GDS, Geriatric Depression Scale.

Diagnostic criteria included DSM-III, DSM-IV, DSM-5, and ICD-10. Measurement of ACEs varied, though the majority assessed physical abuse, sexual abuse, physical neglect, emotional abuse, and emotional neglect, often using validated instruments such as the Childhood Trauma Questionnaire (CTQ).

According to the NOS, nine out of 41 studies showed an overall low risk of bias (total score 8–9), while the risk of bias was moderate (total score 4–7) for the remaining studies.

The meta-analysis of the primary outcome revealed an association between ACEs and suicidal behavior in individuals with any affective disorder (OR 1.98; 95% CI 1.92–2.51; *I*^2^ = 55.5%). According to the Umbrella Review Criteria, the strength of the association was “Highly Suggestive” (Class II). The following shortcomings limited the strength of the association: risk of small-study effects according to Egger’s test (*p* 0. ≤ 10); the presence of an excess of significance bias (*p* ≤ 0.10); and predicted interval including the null value. Subgroups of different diagnoses confirmed a similar risk for individuals with MDD (OR 2.12; 95% CI 1.64–2.63; *I*^2^ = 71.1%), BD (OR 2.20; 95% CI 1.92–2.51; *I*^2^ = 0.0%) and for studies including mixed diagnosis (OR 1.53; 95% CI 1.31–1.78; *I*^2^ = 6.1%).

Sensitivity analyses removing studies for which we used CTQ mean scores to impute the number of individuals exposed and unexposed to ACEs (7 out of 41 studies, 17.1%), for which adjusted ORs were not available, and with a NOS score ≤ 5 provided results that were broadly consistent with the primary analysis., with no relevant changes also in terms of heterogeneity, which remained moderate-to-substantial in the subgroup of individuals with MDD. Meta-regression analyses did not show a moderating effect of mean age, percentages of bipolar individuals, substance abusers, and comorbidity with anxiety disorders and with any mental health condition (see Supplementary Materials). Several pre-planned sensitivity analyses and meta-regressions could not be performed due to the lack of data from original studies.

Secondary outcomes are summarized in Table 2. An increased risk of any suicidal behavior was confirmed for all subtypes of ACEs, namely (ordered by largest to smallest effect size): sexual abuse (OR 2.24; 95% CI 1.90–2.64; *I*^2^ = 31.2%), physical abuse (OR 1.85; 95% CI 1.57–2.17; *I*^2^ = 36.7%), emotional abuse (OR 2.05; 95% CI 1.60–2.63; *I*^2^ = 34.5%), emotional neglect (OR 1.57; 95% CI 1.24–2.00; *I*^2^ = 24.9%), and physical neglect (OR 1.43; 95% CI 1.15–1.78; *I*^2^ = 19.5%). Similarly, an increased risk of suicidal ideation (OR 2.16; 95% CI 1.42–3.29; *I*^2^ = 78.8%) and suicide attempt (OR 1.95; 95% CI 1.70–2.25; *I*^2^ = 54.1%) emerged for people exposed to any ACE, while we could not analyze the risk of death by suicide and NSSI due to lack of data from original studies. Similarly, we were not able to analyze the risk of suicidal behavior as a function of the severity of the ACEs as expressed by the CTQ-SF score.

**Table 2.** Results of secondary analyses

Analysis	No. of studies	No. of participants	Exposed to ACEs	Non-exposed to ACEs	OR (95% CI)	I^2^
Any suicide behavior in individuals exposed to sexual abuse	25	11,037	2,869	8,168	2.42 (1.90–2.64)	31.2%
Any suicide behavior in individuals exposed to physical abuse	22	10,931	2,731	8,200	1.85 (1.57–2.17)	36.7%
Any suicide behavior in individuals exposed to physical neglect	11	2,900	1,293	1,607	1.43 (1.15–1.78)	19.5%
Any suicide behavior in individuals exposed to emotional abuse	11	2,981	1,375	1,606	2.05 (1.60–2.63)	34.5%
Any suicide behavior in individuals exposed to emotional neglect	11	3,095	1,411	1,684	1.57 (1.24–2.00)	24.9%
Suicide ideation in individuals exposed to any ACEs	9	2,482	897	1,585	2.16 (1.42–3.29)	78.8%
Suicide attempt in individuals exposed to any ACEs	33	12,940	4,102	8,838	1.95 (1.70–2.25)	54.1%

## Discussion

To the best of our knowledge, this is the largest and most comprehensive systematic review and meta-analysis exploring the association between ACEs and suicidal behavior in individuals with affective disorders.

Results from our analyses indicate that, among individuals with affective disorders, exposure to ACEs is associated with approximately twice the odds of suicidal behavior; the risk is similar between MDD and BP; the risk remains significant for both suicidal ideation and suicide attempts and for each different type of ACE, with a possibility more substantial effect in those exposed to sexual abuse, although a comparison between different ACEs remains merely exploratory due to the relatively imprecise results for some of them.

Our findings align with prior meta-analytic evidence in the general population, which has consistently reported an increased risk of suicidal behaviors among individuals exposed to ACEs [26]. However, we extend these insights to mood disorders specifically—an area that had been relatively understudied. While Angelakis and colleagues [6] found an approximate two- to three-fold rise in suicide risk in the general population with a history of childhood maltreatment, the present analysis observed a similarly elevated risk (OR ≈ 2.0) among individuals with MD or BD. Our work also resonates with findings from schizophrenia-spectrum studies [15], where ACEs have been robustly linked with higher suicidality rates. Taken together, these parallel results strengthen the broader claim that early-life trauma exerts a transdiagnostic influence on suicide risk, yet our focus on MDD and BD clarifies the magnitude of risk in these specific mood disorder populations.

Moreover, we found that certain forms of abuse—particularly sexual abuse—demonstrated a stronger association with suicidality. This observation builds on a smaller-scale study [67], suggesting that sexual trauma may lead to more persistent mood dysregulation and heightened impulsivity, thus increasing suicide vulnerability. By focusing solely on mood disorders, our meta-analysis highlights the need for ongoing routine assessment of ACEs in these groups and the potential utility of trauma-informed interventions tailored to specific types of early adversity. A key consideration in this study is that data on childhood adversity necessarily rely on observational designs. Due to ethical constraints, one cannot experimentally assign or manipulate ACEs in a controlled trial. Moreover, large-scale observational cohorts provide a real-world perspective that can capture complex presentations and comorbidities in individuals with affective disorders. While these designs carry inherent limitations—such as recall bias and the potential for residual confounding—they remain indispensable for studying potentially harmful exposures like childhood maltreatment.

Finally, it is worth highlighting that while the association between ACEs and suicidal behaviors may appear intuitive, our study contributes novel insights by focusing exclusively on individuals with major depressive disorder and bipolar disorder. By pooling data from multiple designs and examining both suicidal ideation and suicide attempts, this meta-analysis provides the largest and most detailed overview to date of how specific forms of childhood adversity (such as sexual, physical, or emotional abuse) contribute to heightened suicidality in these populations. These findings underscore the importance of systematically evaluating ACEs and highlight the need for targeted intervention strategies in mental health services. In doing so, our work fills a critical gap in the evidence base, offering clinicians and policymakers a more nuanced understanding of how early adverse experiences can influence the trajectories of affective disorders.

This review has several limitations. First, most of the included studies employed a retrospective study design, which might be associated with the risk of recall bias for both exposures to traumatic events and suicidal behavior and, in general, prevent establishing a causal effect. Second, data of interest were often lacking. However, we could meta-analyze data imputed according to validated methodologies for most of them. Although such approaches are based on assumptions (e.g., the clinical relevance of specific rating scale cut-offs), a sensitivity analysis removing imputed data was consistent with the primary analysis. Third, a lack of meta-analyzable data prevented from analyzing clinically relevant outcomes, including deaths by suicide, NSSI, which might often be underestimated and underreported [68, 69], and whether the severity of ACEs might increase the risk of suicidal behavior, as suggested by some of the largest individual studies included [70, 71]. Fourth, although we analyzed different ACE subtypes separately, we are aware that most individuals were probably exposed to multiple co-occurring traumatic experiences [30], which cannot be fully disentangled through subgroup analyses. Further, studies did not report sufficient information on individuals experiencing “complex trauma” to allow for a dedicated analysis. Fifth, only a few of the pre-planned meta-regression analyses were carried out, and, for some of them, relatively few studies contributed, preventing a clear interpretation of the possible role of effect moderators.

Despite these limitations, these results have relevant implications for clinical practice, research, and policy. Routinely screening for ACEs in individuals with affective disorders can provide practical insights into the clinical trajectory and prognosis, including the risk of suicide behaviors. As ACEs might often be unapparent or underreported, a simple validated questionnaire, such as the CTQ-SF, can be effectively implemented with minimal time deduction from ordinary practice. Dedicated training for healthcare professionals can further increase awareness and improve the detection of such an important construct. If relevant ACEs are identified, regular monitoring of co-occurrent risk factors, along with tailored psychosocial support, including trauma-focused psychological interventions, should be provided.

Future large-scale longitudinal studies can elucidate how the timing, progression, and co-occurrence of ACEs influence the onset and severity of suicidal behaviors. They can also provide valuable insights into potential effect moderators, which remain understudied and underreported despite their significant clinical importance. Future advancements in trauma-focused psychotherapies, such as eye-movement desensitization and reprocessing (EMDR) and trauma-focused cognitive-behavioral therapy (TF-CBT), could greatly benefit from these insights [72, 73]. These therapies specifically address and process painful or distressing memories, making them particularly relevant for individuals with a history of ACEs. Our findings underscore both the high prevalence and significant impact of ACEs in individuals with affective disorders, as well as the potentially heightened odds of suicidality associated with specific forms of maltreatment (e.g., sexual abuse). By delineating which types of ACEs are most strongly correlated with suicidal behavior, clinicians can tailor trauma-focused interventions to better address the unique emotional and cognitive patterns arising from these adversities. For example, identifying patients with a history of sexual abuse could prompt the incorporation of EMDR techniques aimed at reprocessing trauma-related cues or self-blaming beliefs. Similarly, TF-CBT modules could be adapted to focus more intensively on maladaptive cognitions and attachment disruptions stemming from emotional abuse. A clearer understanding of the ACE-suicidality relationship will allow mental health professionals to refine these interventions, ultimately contributing to more targeted, evidence-based trauma work with the potential to reduce suicide risk in this vulnerable population. Furthermore, pragmatic randomized-controlled trials recruiting individuals exposed to ACEs can be designed to assess the preventive effect of these psychological interventions on suicidal behaviors.

Regarding policy implications, universal, selected prevention programs targeting vulnerable populations should be implemented to minimize exposure to ACEs. Moreover, prompt access to dedicated healthcare pathways for young, traumatized individuals and offering targeted psychosocial interventions might effectively mitigate the impact of traumatic experiences, reducing, therefore, associated risks on several mental health outcomes, including suicide.

In summary, our meta-analysis provides evidence that exposure to adverse childhood experiences nearly doubles the risk of suicidal behaviors in individuals with affective disorders. Although these findings help fill a gap in understanding the interplay between childhood adversity and suicidality in mood disorders, they should be interpreted in light of the predominantly retrospective and observational nature of the included studies. This design carries an inherent risk of recall bias and residual confounding, which limits the ability to draw definitive causal inferences. Future large-scale, prospective investigations and standardized assessment methods are warranted to corroborate and extend our findings, ultimately guiding trauma-focused preventive and therapeutic strategies in clinical practice.

## Supporting information

Baldini et al. supplementary material Baldini et al. supplementary material

## Data Availability

Data are available upon request from the corresponding author.

## Supplementary material

The supplementary material for this article can be found at http://doi.org/10.1192/j.eurpsy.2025.2452.
