# Supplementary material for: Association between adverse childhood experiences and suicidal behavior in affective disorders: A systematic review and meta-analysis
Source: Eur Psychiatry. 2025 May 28;68(1):e58. doi: 10.1192/j.eurpsy.2025.2452 (PMC12188341; doi:10.1192/j.eurpsy.2025.2452)

Supplemental Data

Baldini et al. Association between adverse childhood experiences and suicidal behavior in affective disorders: a systematic review and meta-analysis

Index of Supplements

1. **PRISMA checklist** p. 2
2. **Search strategy** p. 6
3. **List of excluded studies** p. 9
4. **Risk of bias (Newcastle-Ottawa scale assessment)** p. 14
5. **Data extraction and imputation** p. 16
6. **Primary outcome: any suicidal behavior (any childhood adverse experience)** p. 17 p. 18
   - Umbrella Review Criteria: assessment of the overall credibility of the evidence p. 31 p. 34
   - Sensitivity analyses p. 21
   - Meta-regression analyses p. 25
7. **Secondary analyses** p. 32
   - Severity of ACEs
   - Suicidal ideation (any ACE)
   - Suicide attempt (any ACE)
   - Completed suicide (any ACE)
   - Non-suicidal self-injury (any ACE)
   - Any suicide behavior (physical abuse)
   - Any suicide behavior (physical neglect)
   - Any suicide behavior (emotional abuse)
   - Any suicide behavior (emotional neglect)
   - Any suicide behavior (sexual abuse)

Supplement 1. PRISMA checklist

Section and Topic	Item #	Checklist item	Location where item is reported
**TITLE**			
Title	1	Identify the report as a systematic review.	p.1
**ABSTRACT**			
Abstract	2	See the PRISMA 2020 for Abstracts checklist.	p.2
**INTRODUCTION**			
Rationale	3	Describe the rationale for the review in the context of existing knowledge.	p.3
Objectives	4	Provide an explicit statement of the objective(s) or question(s) the review addresses.	p.4
**METHODS**			
Eligibility criteria	5	Specify the inclusion and exclusion criteria for the review and how studies were grouped for the syntheses.	p.4-5
Information sources	6	Specify all databases, registers, websites, organisations, reference lists and other sources searched or consulted to identify studies. Specify the date when each source was last searched or consulted.	p.5
Search strategy	7	Present the full search strategies for all databases, registers and websites, including any filters and limits used.	Supplement 2 p.6-8
Selection process	8	Specify the methods used to decide whether a study met the inclusion criteria of the review, including how many reviewers screened each record and each report retrieved, whether they worked independently, and if applicable, details of automation tools used in the process.	p.5
Data collection process	9	Specify the methods used to collect data from reports, including how many reviewers collected data from each report, whether they worked independently, any processes for obtaining or confirming data from study investigators, and if applicable, details of automation tools used in the process.	p.6
Data items	10a	List and define all outcomes for which data were sought. Specify whether all results that were compatible with each outcome domain in each study were sought (e.g. for all measures, time points, analyses), and if not, the methods used to decide which results to collect.	p.8
	10b	List and define all other variables for which data were sought (e.g. participant and intervention	p.11

Section and Topic	Item #	Checklist item	Location where item is reported
		characteristics, funding sources). Describe any assumptions made about any missing or unclear information.	
Study risk of bias assessment	11	Specify the methods used to assess risk of bias in the included studies, including details of the tool(s) used, how many reviewers assessed each study and whether they worked independently, and if applicable, details of automation tools used in the process.	p.6
Effect measures	12	Specify for each outcome the effect measure(s) (e.g. risk ratio, mean difference) used in the synthesis or presentation of results.	p.9
Synthesis methods	13a	Describe the processes used to decide which studies were eligible for each synthesis (e.g. tabulating the study intervention characteristics and comparing against the planned groups for each synthesis (item #5)).	Supplement 5 p.15-29
	13b	Describe any methods required to prepare the data for presentation or synthesis, such as handling of missing summary statistics, or data conversions.	p.9
	13c	Describe any methods used to tabulate or visually display results of individual studies and syntheses.	Supplement 5 p.15-29
	13d	Describe any methods used to synthesize results and provide a rationale for the choice(s). If meta-analysis was performed, describe the model(s), method(s) to identify the presence and extent of statistical heterogeneity, and software package(s) used.	p.10
	13e	Describe any methods used to explore possible causes of heterogeneity among study results (e.g. subgroup analysis, meta-regression).	Table 2 p.29 and Supplement 6 p.35-42
	13f	Describe any sensitivity analyses conducted to assess robustness of the synthesized results.	Supplement 6 p.33-34
Reporting bias assessment	14	Describe any methods used to assess risk of bias due to missing results in a synthesis (arising from reporting biases).	Supplement 4 p.13-14
Certainty assessment	15	Describe any methods used to assess certainty (or confidence) in the body of evidence for an outcome.	p.11-12
**RESULTS**			
Study selection	16a	Describe the results of the search and selection process, from the number of records identified in the search to the number of studies included in the review, ideally using a flow diagram.	p.11

Section and Topic	Item #	Checklist item	Location where item is reported
	16b	Cite studies that might appear to meet the inclusion criteria, but which were excluded, and explain why they were excluded.	Supplement 3 p.7-12
Study characteristics	17	Cite each included study and present its characteristics.	Table 1 p. 28
Risk of bias in studies	18	Present assessments of risk of bias for each included study.	Supplement 4 p.13-14
Results of individual studies	19	For all outcomes, present, for each study: (a) summary statistics for each group (where appropriate) and (b) an effect estimate and its precision (e.g. confidence/credible interval), ideally using structured tables or plots.	Supplement 6 p.30
Results of syntheses	20a	For each synthesis, briefly summarise the characteristics and risk of bias among contributing studies.	p.13
	20b	Present results of all statistical syntheses conducted. If meta-analysis was done, present for each the summary estimate and its precision (e.g. confidence/credible interval) and measures of statistical heterogeneity. If comparing groups, describe the direction of the effect.	Supplement 6 p.30
	20c	Present results of all investigations of possible causes of heterogeneity among study results.	p.11-12
	20d	Present results of all sensitivity analyses conducted to assess the robustness of the synthesized results.	Supplement 6 p.33-38
Reporting biases	21	Present assessments of risk of bias due to missing results (arising from reporting biases) for each synthesis assessed.	Supplement 4 p.13-14
Certainty of evidence	22	Present assessments of certainty (or confidence) in the body of evidence for each outcome assessed.	p.11-12
**DISCUSSION**			
Discussion	23a	Provide a general interpretation of the results in the context of other evidence.	p.7-8
	23b	Discuss any limitations of the evidence included in the review.	p.8-9
	23c	Discuss any limitations of the review processes used.	p.8-9
	23d	Discuss implications of the results for practice, policy, and future research.	p.9
**OTHER INFORMATION**			
Registration and protocol	24a	Provide registration information for the review, including register name and registration number, or state that the review was not registered.	p.7

Section and Topic	Item #	Checklist item	Location where item is reported
	24b	Indicate where the review protocol can be accessed, or state that a protocol was not prepared.	p.7
	24c	Describe and explain any amendments to information provided at registration or in the protocol.	p.7
Support	25	Describe sources of financial or non-financial support for the review, and the role of the funders or sponsors in the review.	p.16
Competing interests	26	Declare any competing interests of review authors.	p.16
Availability of data, code and other materials	27	Report which of the following are publicly available and where they can be found: template data collection forms; data extracted from included studies; data used for all analyses; analytic code; any other materials used in the review.	Supplement 5 p.15-29

Supplement 2. Search strategy

PubMed (22/05/2023) n=5682
(("mood disorder*"[Title/Abstract] OR "depressive disorder"[Title/Abstract] OR "depress*"[Title/Abstract] OR "affective disorder"[Title/Abstract] OR "affective psychosis"[Title/Abstract] OR "bipolar"[Title/Abstract] OR "manic-depressive"[Title/Abstract] OR "manic"[Title/Abstract]) AND ("self injurious behaviour"[Title/Abstract] OR "self injurious behavior"[MeSH Terms] OR ("self injurious"[Title/Abstract] AND "behavior"[Title/Abstract]) OR "self injurious behavior"[Title/Abstract] OR ("self"[Title/Abstract] AND "injurious"[Title/Abstract] AND "behavior"[Title/Abstract]) OR "self injurious behavior"[Title/Abstract] OR ("suicid"[Title/Abstract] OR "suicidal ideation"[MeSH Terms] OR ("suicidal"[Title/Abstract] AND "ideation"[Title/Abstract]) OR "suicidal ideation"[Title/Abstract] OR "suicidality"[Title/Abstract] OR "suicidal"[Title/Abstract] OR "suicidally"[Title/Abstract] OR "suicidals"[Title/Abstract] OR "suicide"[MeSH Terms] OR "suicide"[Title/Abstract] OR "suicides"[Title/Abstract] OR "suicide s"[Title/Abstract] OR "suicided"[Title/Abstract] OR "suiciders"[Title/Abstract]))) AND ((("adverse"[Title/Abstract] OR "adversely"[Title/Abstract] OR "adverses"[Title/Abstract]) AND ("childhood"[Title/Abstract] OR "childhoods"[Title/Abstract]) AND "experience*"[Title/Abstract]) OR (("childhood"[Title/Abstract] OR "childhoods"[Title/Abstract]) AND "trauma*"[Title/Abstract]) OR (("traumatic"[Title/Abstract] OR "traumatically"[Title/Abstract] OR "traumatism"[Title/Abstract] OR "traumatisms"[Title/Abstract] OR "traumatization"[Title/Abstract] OR "traumatizations"[Title/Abstract] OR "traumatize"[Title/Abstract] OR "traumatized"[Title/Abstract] OR "traumatizes"[Title/Abstract] OR "traumatizing"[Title/Abstract]) AND ("childhood"[Title/Abstract] OR "childhoods"[Title/Abstract]) AND "experience*"[Title/Abstract]) OR (("childhood"[Title/Abstract] OR "childhoods"[Title/Abstract]) AND "experience*"[Title/Abstract]) OR (("adolescences"[Title/Abstract] OR "adolescency"[Title/Abstract] OR "adolescent"[MeSH Terms] OR "adolescent"[Title/Abstract] OR "adolescence"[Title/Abstract] OR "adolescents"[Title/Abstract] OR "adolescent s"[Title/Abstract]) AND "trauma*"[Title/Abstract]) OR ("adverse childhood experiences"[MeSH Terms] OR ("adverse"[Title/Abstract] AND "childhood"[Title/Abstract] AND "experiences"[Title/Abstract]) OR "adverse childhood experiences"[Title/Abstract] OR ("early"[Title/Abstract] AND "life"[Title/Abstract] AND "stress"[Title/Abstract]) OR "early life stress"[Title/Abstract]) OR ("adverse childhood experiences"[MeSH Terms] OR ("adverse"[Title/Abstract] AND "childhood"[Title/Abstract] AND "experiences"[Title/Abstract]) OR "adverse childhood experiences"[Title/Abstract] OR ("early"[Title/Abstract] AND "life"[Title/Abstract] AND "stress"[Title/Abstract]) OR "early life stress"[Title/Abstract]) OR ("sex offenses"[MeSH Terms] OR ("sex"[Title/Abstract] AND "offenses"[Title/Abstract]) OR "sex offenses"[Title/Abstract] OR ("sexual"[Title/Abstract] AND "abuse"[Title/Abstract]) OR "sexual abuse"[Title/Abstract]) OR ("violence"[MeSH Terms] OR "violence"[Title/Abstract] OR "violence s"[Title/Abstract] OR "violences"[Title/Abstract]) OR (("verbal"[Title/Abstract] OR "verbalization"[Title/Abstract] OR "verbalizations"[Title/Abstract] OR "verbalize"[Title/Abstract] OR "verbalized"[Title/Abstract] OR "verbalizer"[Title/Abstract] OR "verbalizers"[Title/Abstract] OR "verbalizing"[Title/Abstract] OR "verbally"[Title/Abstract]) AND ("abusable"[Title/Abstract] OR "abuse s"[Title/Abstract] OR "abused"[Title/Abstract] OR "abuser"[Title/Abstract] OR "abuser s"[Title/Abstract] OR "abusers"[Title/Abstract] OR "abuses"[Title/Abstract] OR "abusing"[Title/Abstract] OR "abusive"[Title/Abstract] OR "abusively"[Title/Abstract] OR "abusiveness"[Title/Abstract] OR "substance related disorders"[MeSH Terms] OR ("substance

related"[Title/Abstract] AND "disorders"[Title/Abstract]) OR "substance related disorders"[Title/Abstract] OR "abuse"[Title/Abstract])) OR ("emotional abuse"[MeSH Terms] OR ("emotional"[Title/Abstract] AND "abuse"[Title/Abstract]) OR "emotional abuse"[Title/Abstract]) OR ("abusable"[Title/Abstract] OR "abuse s"[Title/Abstract] OR "abused"[Title/Abstract] OR "abuser"[Title/Abstract] OR "abuser s"[Title/Abstract] OR "abusers"[Title/Abstract] OR "abuses"[Title/Abstract] OR "abusing"[Title/Abstract] OR "abusive"[Title/Abstract] OR "abusively"[Title/Abstract] OR "abusiveness"[Title/Abstract] OR "substance related disorders"[MeSH Terms] OR ("substance related"[Title/Abstract] AND "disorders"[Title/Abstract]) OR "substance related disorders"[Title/Abstract] OR "abuse"[Title/Abstract]) OR ("neglect"[Title/Abstract] OR "neglected"[Title/Abstract] OR "neglectful"[Title/Abstract] OR "neglecting"[Title/Abstract] OR "neglects"[Title/Abstract]) OR (("parent s"[Title/Abstract] OR "parentally"[Title/Abstract] OR "parentals"[Title/Abstract] OR "parented"[Title/Abstract] OR "parenting"[MeSH Terms] OR "parenting"[Title/Abstract] OR "parents"[MeSH Terms] OR "parents"[Title/Abstract] OR "parent"[Title/Abstract] OR "parental"[Title/Abstract]) AND ("neglect"[Title/Abstract] OR "neglected"[Title/Abstract] OR "neglectful"[Title/Abstract] OR "neglecting"[Title/Abstract] OR "neglects"[Title/Abstract])) OR (("physical examination"[MeSH Terms] OR ("physical"[Title/Abstract] AND "examination"[Title/Abstract]) OR "physical examination"[Title/Abstract] OR "physical"[Title/Abstract] OR "physically"[Title/Abstract] OR "physicals"[Title/Abstract]) AND ("neglect"[Title/Abstract] OR "neglected"[Title/Abstract] OR "neglectful"[Title/Abstract] OR "neglecting"[Title/Abstract] OR "neglects"[Title/Abstract])) OR (("emoting"[Title/Abstract] OR "emotion s"[Title/Abstract] OR "emotions"[MeSH Terms] OR "emotions"[Title/Abstract] OR "emotion"[Title/Abstract] OR "emotional"[Title/Abstract] OR "emotive"[Title/Abstract]) AND ("neglect"[Title/Abstract] OR "neglected"[Title/Abstract] OR "neglectful"[Title/Abstract] OR "neglecting"[Title/Abstract] OR "neglects"[Title/Abstract])) OR ("bullying"[MeSH Terms] OR "bullying"[Title/Abstract] OR "bullied"[Title/Abstract] OR "bullies"[Title/Abstract]) OR (("parent s"[Title/Abstract] OR "parentally"[Title/Abstract] OR "parentals"[Title/Abstract] OR "parented"[Title/Abstract] OR "parenting"[MeSH Terms] OR "parenting"[Title/Abstract] OR "parents"[MeSH Terms] OR "parents"[Title/Abstract] OR "parent"[Title/Abstract] OR "parental"[Title/Abstract]) AND "loss"[Title/Abstract]) OR "divorce"[Title/Abstract])) AND (adolescent[Filter] OR alladult[Filter])

CINHAL 22/05/2023 n=318
(AB (self injurious behaviour OR self injur* OR self-injur* OR self mutilation OR self-harm OR self harm OR Nonsuicidal Self Injury OR Self-Destructive Behavior OR suicide* OR attempted suicide OR suicide attempt* OR suicidal ideation OR suicidal behavior OR suicidal*) AND AB (Adverse Childhood Experience* OR Childhood Trauma* OR Traumatic Childhood Experience* OR Childhood Experience* OR Adolescent Trauma* OR Early Life Stress OR Early-Life Stress OR sexual abuse OR violence OR verbal abuse OR emotional abuse OR abuse OR neglect OR parental neglect OR physical neglect OR emotional neglect OR bullying OR parental loss OR divorce) AND AB (mood disorder OR depressive disorder OR depress* OR affective disorder OR affective psychosis OR bipolar OR manic-depressive OR manic).) NOT TI review NOT TI random*
Limiters - Published Date: 1990-current; Narrow by SubjectAge: - adolescent: 13-18 years & all adult

PsychINFO 22/05/2023 n=2115

((self injurious behaviour or self injur* or self-injur* or self mutilation or self-harm or self harm or Nonsuicidal Self Injury or Self-Destructive Behavior or suicide* or attempted suicide or suicide attempt* or suicidal ideation or suicidal behavior or suicidal*) and (Adverse Childhood Experience* or Childhood Trauma* or Traumatic Childhood Experience* or Childhood Experience* or Adolescent Trauma* or Early Life Stress or Early-Life Stress or sexual abuse or violence or verbal abuse or emotional abuse or abuse or neglect or parental neglect or physical neglect or emotional neglect or bullying or parental loss or divorce) and (mood disorder or depressive disorder or depress* or affective disorder or affective psychosis or bipolar or manic-depressive or manic)).ab.
Limit to (human and english language and ("0200 clinical case study" or "0400 empirical study" or "0430 followup study" or "0450 longitudinal study" or "0451 prospective study" or "0453 retrospective study") and adulthood <18+ years> and "0110 peer-reviewed journal" and journal article and yr="1990 -Current")

Web of Science Core Collection 22/05/2023 n=2030
Topic= self injurious behaviour OR self injur* OR self-injur* OR self mutilation OR self-harm OR self harm OR Nonsuicidal Self Injury OR Self-Destructive Behavior OR suicide* OR attempted suicide OR suicide attempt* OR suicidal ideation OR suicidal behavior OR suicidal*
AND
Title= Adverse Childhood Experience* OR Childhood Trauma* OR Traumatic Childhood Experience* OR Childhood Experience* OR Adolescent Trauma* OR Early Life Stress OR Early-Life Stress OR sexual abuse OR violence OR verbal abuse OR emotional abuse OR abuse OR neglect OR parental neglect OR physical neglect OR emotional neglect OR bullying OR parental loss OR divorce
AND
Topic= mood disorder OR depressive disorder OR depress* OR affective disorder OR affective psychosis OR bipolar OR manic-depressive OR manic

Supplement 3. List of excluded studies

We selected 82 records for potential inclusion and evaluated them for eligibility by examining the study's full text. 41 records were included in the systematic review, and the remaining 41 records were excluded. The reasons for exclusion were:

Unable to obtain sufficient data

1- Adigüzel V, Özdemir N, Şahin ŞK. Childhood traumas in euthymic bipolar disorder patients in Eastern Turkey and its relations with suicide risk and aggression. Nord J Psychiatry. 2019 Nov;73(8):490-496. doi: 10.1080/08039488.2019.1655589. Epub 2019 Aug 29. PMID: 31464549.
2- Lamis DA, Kapoor S, Evans APB. Childhood Sexual Abuse and Suicidal Ideation Among Bipolar Patients: Existential But Not Religious Well-Being as a Protective Factor. Suicide Life Threat Behav. 2019 Apr;49(2):401-412. doi: 10.1111/sltb.12438. Epub 2018 Feb 12. PMID: 29430741.
3- Moraes JB, Maes M, Roomruangwong C, Bonifacio KL, Barbosa DS, Vargas HO, Anderson G, Kubera M, Carvalho AF, Nunes SOV. In major affective disorders, early life trauma predict increased nitro-oxidative stress, lipid peroxidation and protein oxidation and recurrence of major affective disorders, suicidal behaviors and a lowered quality of life. Metab Brain Dis. 2018 Aug;33(4):1081-1096. doi: 10.1007/s11011-018-0209-3. Epub 2018 Mar 14. PMID: 29542039.
4- Mazer AK, Cleare AJ, Young AH, Juruena MF. Bipolar affective disorder and borderline personality disorder: Differentiation based on the history of early life stress and psychoneuroendocrine measures. Behav Brain Res. 2019 Jan 14;357-358:48-56. doi: 10.1016/j.bbr.2018.04.015. Epub 2018 Apr 24. PMID: 29702176.
5- Singh AB, Bousman CA, Ng CH, Berk M. High impact child abuse may predict risk of elevated suicidality during antidepressant initiation. Aust N Z J Psychiatry. 2013 Dec;47(12):1191-5. doi: 10.1177/0004867413510212. PMID: 24280998.
6- Pompili M, Erbuto D, Innamorati M, Luciano M, Sampogna G, Abbate-Daga G, Barlati S, Carmassi C, Castellini G, De Fazio P, Di Lorenzo G, Di Nicola M, Ferrari S, Gramaglia C, Nanni MG, Pasquini M, Pinna F, Poloni N, Serafini G, Signorelli M, Ventriglio A, Volpe U, Fiorillo A. The Relationship Between Mental Pain, Suicide Risk, and Childhood Traumatic Experiences: Results From a Multicenter Study. J Clin Psychiatry. 2022 Jun 15;83(4):21m14176. doi: 10.4088/JCP.21m14176. PMID: 35704708.
7- Vitriol V, Cancino A, Leiva-Bianchi M, Serrano C, Ballesteros S, Potthoff S, Cáceres C, Ormazábal M, Asenjo A. Depresión adulta y experiencias infantiles adversas: evidencia de un subtipo depresivo complejo en consultantes de la atención primaria en Chile [Association between adverse childhood experiences with depression in adults consulting in primary care]. Rev Med Chil. 2017 Sep;145(9):1145-1153. Spanish. doi: 10.4067/s0034-98872017000901145. PMID: 29424401.
8- Pompili M, Innamorati M, Lamis DA, Erbuto D, Venturini P, Ricci F, Serafini G, Amore M, Girardi P. The associations among childhood maltreatment, "male depression" and suicide risk in psychiatric patients. Psychiatry Res. 2014 Dec 15;220(1-2):571-8. doi: 10.1016/j.psychres.2014.07.056. Epub 2014 Aug 13. PMID: 25169890.
9- Xie P, Wu K, Zheng Y, Guo Y, Yang Y, He J, Ding Y, Peng H. Prevalence of childhood trauma and correlations between childhood trauma, suicidal ideation, and social support in patients with depression, bipolar disorder, and schizophrenia in southern

China. J Affect Disord. 2018 Mar 1;228:41-48. doi: 10.1016/j.jad.2017.11.011. Epub 2017 Nov 6. PMID: 29223913.

10- Breen ME, Seifuddin F, Zandi PP, Potash JB, Willour VL. Investigating the role of early childhood abuse and HPA axis genes in suicide attempters with bipolar disorder. Psychiatr Genet. 2015 Jun;25(3):106-11. doi: 10.1097/YPG.0000000000000082. PMID: 25714448; PMCID: PMC4412784.

Inappropriate study design

Wrong population

1- Macalli M, Orri M, Tzourio C, Côté SM. Contributions of childhood peer victimization and/or maltreatment to young adult anxiety, depression, and suicidality: a cross-sectional study. BMC Psychiatry. 2021 Jul 14;21(1):354. doi: 10.1186/s12888-021-03354-4. PMID: 34261482; PMCID: PMC8278608.
2- Cohen Y, Spirito A, Sterling C, Donaldson D, Seifer R, Plummer B, Avila R, Ferrer K. Physical and sexual abuse and their relation to psychiatric disorder and suicidal behavior among adolescents who are psychiatrically hospitalized. J Child Psychol Psychiatry. 1996 Nov;37(8):989-93. doi: 10.1111/j.1469-7610.1996.tb01495.x. PMID: 9119945.
3- Gawęda Ł, Pionke R, Krężołek M, Frydecka D, Nelson B, Cechnicki A. The interplay between childhood trauma, cognitive biases, psychotic-like experiences and depression and their additive impact on predicting lifetime suicidal behavior in young adults. Psychol Med. 2020 Jan;50(1):116-124. doi: 10.1017/S0033291718004026. Epub 2019 Jan 10. PMID: 30626466.
4- He Y, Zhang Y, Cui X, Zhong Y, He W, Liu J, Luo X, Gong J. Epidemiology of major childhood adversities and its effect on depression and suicide attempts in Chinese college students. J Affect Disord. 2021 Feb 15;281:331-337. doi: 10.1016/j.jad.2020.12.031. Epub 2020 Dec 8. PMID: 33341646.
5- Stewart JG, Kim JC, Esposito EC, Gold J, Nock MK, Auerbach RP. Predicting suicide attempts in depressed adolescents: Clarifying the role of disinhibition and childhood sexual abuse. J Affect Disord. 2015 Nov 15;187:27-34. doi: 10.1016/j.jad.2015.08.034. Epub 2015 Aug 19. PMID: 26318268; PMCID: PMC4587293.
6- Brockie TN, Dana-Sacco G, Wallen GR, Wilcox HC, Campbell JC. The Relationship of Adverse Childhood Experiences to PTSD, Depression, Poly-Drug Use and Suicide Attempt in Reservation-Based Native American Adolescents and Young Adults. Am J Community Psychol. 2015 Jun;55(3-4):411-21. doi: 10.1007/s10464-015-9721-3. PMID: 25893815.
7- Brand EF, King CA, Olson E, Ghaziuddin N, Naylor M. Depressed adolescents with a history of sexual abuse: diagnostic comorbidity and suicidality. J Am Acad Child Adolesc Psychiatry. 1996 Jan;35(1):34-41. doi: 10.1097/00004583-199601000-00010. PMID: 8567608.
8- Caravaca Sánchez F, Ignatyev Y, Mundt AP. Associations between childhood abuse, mental health problems, and suicide risk among male prison populations in Spain. Crim Behav Ment Health. 2019 Feb;29(1):18-30. doi: 10.1002/cbm.2099. Epub 2018 Dec 18. PMID: 30561144.
9- Dunn EC, McLaughlin KA, Slopen N, Rosand J, Smoller JW. Developmental timing of child maltreatment and symptoms of depression and suicidal ideation in young adulthood: results from the National Longitudinal Study of Adolescent Health. Depress

Anxiety. 2013 Oct;30(10):955-64. doi: 10.1002/da.22102. Epub 2013 Apr 16. PMID: 23592532; PMCID: PMC3873604.

10- Andreu Pascual M, Levenson JC, Merranko J, Gill MK, Hower H, Yen S, Strober M, Goldstein TR, Goldstein BI, Ryan ND, Weinstock LM, Keller MB, Axelson D, Birmaher B. The Effect of Traumatic Events on the Longitudinal Course and Outcomes of Youth with Bipolar Disorder. J Affect Disord. 2020 Sep 1;274:126-135. doi: 10.1016/j.jad.2020.05.131. Epub 2020 May 22. PMID: 32469795; PMCID: PMC7365761.

11- Cankaya B, Talbot NL, Ward EA, Duberstein PR. Parental sexual abuse and suicidal behaviour among women with major depressive disorder. Can J Psychiatry. 2012 Jan;57(1):45-51. doi: 10.1177/070674371205700108. PMID: 22296967; PMCID: PMC3417314.

12- Schönfelder A, Hallensleben N, Spangenberg L, Forkmann T, Rath D, Glaesmer H. The role of childhood abuse for suicidality in the context of the interpersonal theory of suicide: An investigation in German psychiatric inpatients with depression. J Affect Disord. 2019 Feb 15;245:788-797. doi: 10.1016/j.jad.2018.11.063. Epub 2018 Nov 5. PMID: 30448764.

13- Smith NB, Monteith LL, Rozek DC, Meuret AE. Childhood Abuse, the Interpersonal-Psychological Theory of Suicide, and the Mediating Role of Depression. Suicide Life Threat Behav. 2018 Oct;48(5):559-569. doi: 10.1111/sltb.12380. Epub 2017 Oct 25. PMID: 29068069.

14- Youssef NA, Green KT, Dedert EA, Hertzberg JS, Calhoun PS, Dennis MF; Mid-Atlantic Mental Illness Research Education And Clinical Center Workgroup; Beckham JC. Exploration of the influence of childhood trauma, combat exposure, and the resilience construct on depression and suicidal ideation among U.S. Iraq/Afghanistan era military personnel and veterans. Arch Suicide Res. 2013;17(2):106-22. doi: 10.1080/13811118.2013.776445. PMID: 23614484; PMCID: PMC3640796.

15- Dervic K, Grunebaum MF, Burke AK, Mann JJ, Oquendo MA. Protective factors against suicidal behavior in depressed adults reporting childhood abuse. J Nerv Ment Dis. 2006 Dec;194(12):971-4. doi: 10.1097/01.nmd.0000243764.56192.9c. PMID: 17164639.

16- Nrugham L, Holen A, Sund AM. Associations between attempted suicide, violent life events, depressive symptoms, and resilience in adolescents and young adults. J Nerv Ment Dis. 2010 Feb;198(2):131-6. doi: 10.1097/NMD.0b013e3181cc43a2. Erratum in: J Nerv Ment Dis. 2010 May;198(5):389. PMID: 20145488.

17- Kivelä L, Krause-Utz A, Mouthaan J, Schoorl M, de Kleine R, Elzinga B, Eikelenboom M, Penninx BW, van der Does W, Antypa N. Longitudinal course of suicidal ideation and predictors of its persistence - A NESDA study. J Affect Disord. 2019 Oct 1;257:365-375. doi: 10.1016/j.jad.2019.07.042. Epub 2019 Jul 5. PMID: 31302526.

18- Athey A, Overholser JC, Beale EE. Depressed adolescents' exposure to suicide attempts and suicide loss. Death Stud. 2022;46(8):1862-1869. doi: 10.1080/07481187.2020.1864063. Epub 2021 Jan 13. PMID: 33439771.

19- Easton SD, Renner LM, O'Leary P. Suicide attempts among men with histories of child sexual abuse: examining abuse severity, mental health, and masculine norms. Child Abuse Negl. 2013 Jun;37(6):380-7. doi: 10.1016/j.chiabu.2012.11.007. Epub 2013 Jan 11. PMID: 23313078.

20- Kamali M, Reilly-Harrington NA, Chang WC, McInnis M, McElroy SL, Ketter TA, Shelton RC, Deckersbach T, Tohen M, Kocsis JH, Calabrese JR, Gao K, Thase ME, Bowden CL, Kinrys G, Bobo WV, Brody BD, Sylvia LG, Rabideau DJ, Nierenberg AA. Bipolar

depression and suicidal ideation: Moderators and mediators of a complex relationship. J Affect Disord. 2019 Dec 1;259:164-172. doi: 10.1016/j.jad.2019.08.032. Epub 2019 Aug 19. PMID: 31445343.

21- Lemieux S, Tourigny M, Joly J, Baril K, Séguin M. Caractéristiques associées à la dépression et aux symptômes de stress post-traumatique chez les femmes victimes d'agression sexuelle durant l'enfance [Characteristics associated with depression and post-traumatic stress disorder among childhood sexual abuse women]. Rev Epidemiol Sante Publique. 2019 Sep;67(5):285-294. French. doi: 10.1016/j.respe.2019.05.012. Epub 2019 Jul 25. PMID: 31353238.
22- Zhang Y, Hu Z, Hu M, Lu Z, Yu H, Yuan X. Effects of childhood trauma on nonsuicidal self-injury in adolescent patients with bipolar II depression. Brain Behav. 2022 Nov;12(11):e2771. doi: 10.1002/brb3.2771. Epub 2022 Sep 28. PMID: 36168882; PMCID: PMC9660408.
23- Hart SR, Van Eck K, Ballard ED, Musci RJ, Newcomer A, Wilcox HC. Subtypes of suicide attempters based on longitudinal childhood profiles of co-occurring depressive, anxious and aggressive behavior symptoms. Psychiatry Res. 2017 Nov;257:150-155. doi: 10.1016/j.psychres.2017.07.032. Epub 2017 Jul 20. PMID: 28755606.

Wrong exposure

1- Aas M, Bellivier F, Bettella F, Henry C, Gard S, Kahn JP, Lagerberg TV, Aminoff SR, Melle I, Leboyer M, Jamain S, Andreassen OA, Etain B. Childhood maltreatment and polygenic risk in bipolar disorders. Bipolar Disord. 2020 Mar;22(2):174-181. doi: 10.1111/bdi.12851. Epub 2019 Nov 13. PMID: 31628696.
2- Aas M, Henry C, Bellivier F, Lajnef M, Gard S, Kahn JP, Lagerberg TV, Aminoff SR, Bjella T, Leboyer M, Andreassen OA, Melle I, Etain B. Affective lability mediates the association between childhood trauma and suicide attempts, mixed episodes and co-morbid anxiety disorders in bipolar disorders. Psychol Med. 2017 Apr;47(5):902-912. doi: 10.1017/S0033291716003081. Epub 2016 Nov 29. PMID: 27894372.

No outcome of interest

1- Aubert E, Jaussent I, Olié E, Ducasse D, Azorin JM, Bellivier F, Belzeaux R, Bougerol T, Etain B, Gard S, Henry C, Kahn JP, Leboyer M, Loftus J, Passerieux C, Lopez-Castroman J, Courtet P; FondaMental Advanced Centers of Expertise in Bipolar Disorders (FACE-BD) Collaborators. Effect of early trauma on the sleep quality of euthymic bipolar patients. J Affect Disord. 2016 Dec;206:261-267. doi: 10.1016/j.jad.2016.07.045. Epub 2016 Jul 27. PMID: 27517134.
2- Dias de Mattos Souza L, Lopez Molina M, Azevedo da Silva R, Jansen K. History of childhood trauma as risk factors to suicide risk in major depression. Psychiatry Res. 2016 Dec 30;246:612-616. doi: 10.1016/j.psychres.2016.11.002. Epub 2016 Nov 3. PMID: 27825790.
3- Olgiati P, Serretti A. Post-traumatic stress disorder and childhood emotional abuse are markers of subthreshold bipolarity and worse treatment outcome in major depressive disorder. Int Clin Psychopharmacol. 2022 Jan 1;37(1):1-8. doi: 10.1097/YIC.0000000000000380. PMID: 34686642; PMCID: PMC9648980.

Supplement 4. Risk of bias (Newcastle-Ottawa scale assessment)

First author, year	Selection (max 4 stars)	Comparability (max 2 stars)	Exposure/ Outcome (max 3 stars)	NOS Total score (max 9 stars)
Alacreu-Crespo, 2022	4	2	3	9
Andover, 2007	4	2	3	9
Behr Gomes Jardim, 2018	3	2	3	8
Bernegger, 2015	4	2	3	9
Brodsky, 2001	4	2	3	9
Brown, 2005	3	2	3	8
Cakir, 2016	2	0	2	4
Carballo, 2008	4	1	3	8
Chen, 2014	4	2	2	8
Courtet, 2015	3	2	1	6
Etain, 2013	4	1	3	8
Farias, 2019	3	2	3	8
Fijtman, 2023	4	2	3	9
Freitag, 2022	3	2	3	8
Garno, 2005	4	2	3	9
Giampetruzzi, 2023	4	1	3	8
Gladstone, 2004	4	2	3	9
Guillen-Burgos, 2023	3	2	3	8
Horesh, 2003	4	2	3	9
Janiri, 2015	4	2	3	9
Johnstone, 2015	4	1	3	8
Kamali, 2019	4	2	3	9
Kim, 2013	4	2	2	8
Leverich, 2012	4	1	3	8
Lu, 2008	2	2	3	7
McGrady, 2017	4	2	3	9
McIntyre, 2008	4	2	3	9
Miola, 2023	4	2	2	8
Monteleone, 2020	4	1	3	8
Ortiz-Guzman, 2018	4	1	3	8
Perich, 2014	3	2	3	8
Rios, 2020	4	2	3	9
Sarchiapone, 2007	4	2	3	9
Shinozaki, 2013	4	1	3	8
Spinhoven, 2009	4	1	3	8
Talbot, 2004	4	1	3	8

Visioli, 2023	4	2	3	9
Wang, 2021	4	1	3	8
Zlotnik, 2001	4	1	3	8

Supplement 5. Data extraction and imputation

We hereby report a thorough list of the data extracted and imputed for each study contributing to the primary analysis.

For many of the included studies, authors provided the mean scores and standard deviations of the Childhood Trauma Questionnaire (CTQ) (or other rating scales for childhood trauma) for "events" (i.e., individuals with suicide behavior) and "non-events." Considering validated cut-off scores indicating clinically relevant traumatic events (namely, overall score ≥ 35; physical neglect ≥ 8; physical abuse ≥ 8; emotional neglect ≥ 15; emotional abuse ≥ 10; sexual abuse ≥ 8), we employed a validated methodology [5] to detect the number of participants exposed to ACEs within events and non-events.

Whether the study reported only some types of ACEs (e.g., physical abuse, sexual abuse, etc.), we included in the primary analysis the one with the highest number of events (i.e., suicidal behavior) in those exposed to ACEs.

Supplement

Primary outcome: any ACE, any suicidal behavior (subgroups according to the diagnosis)

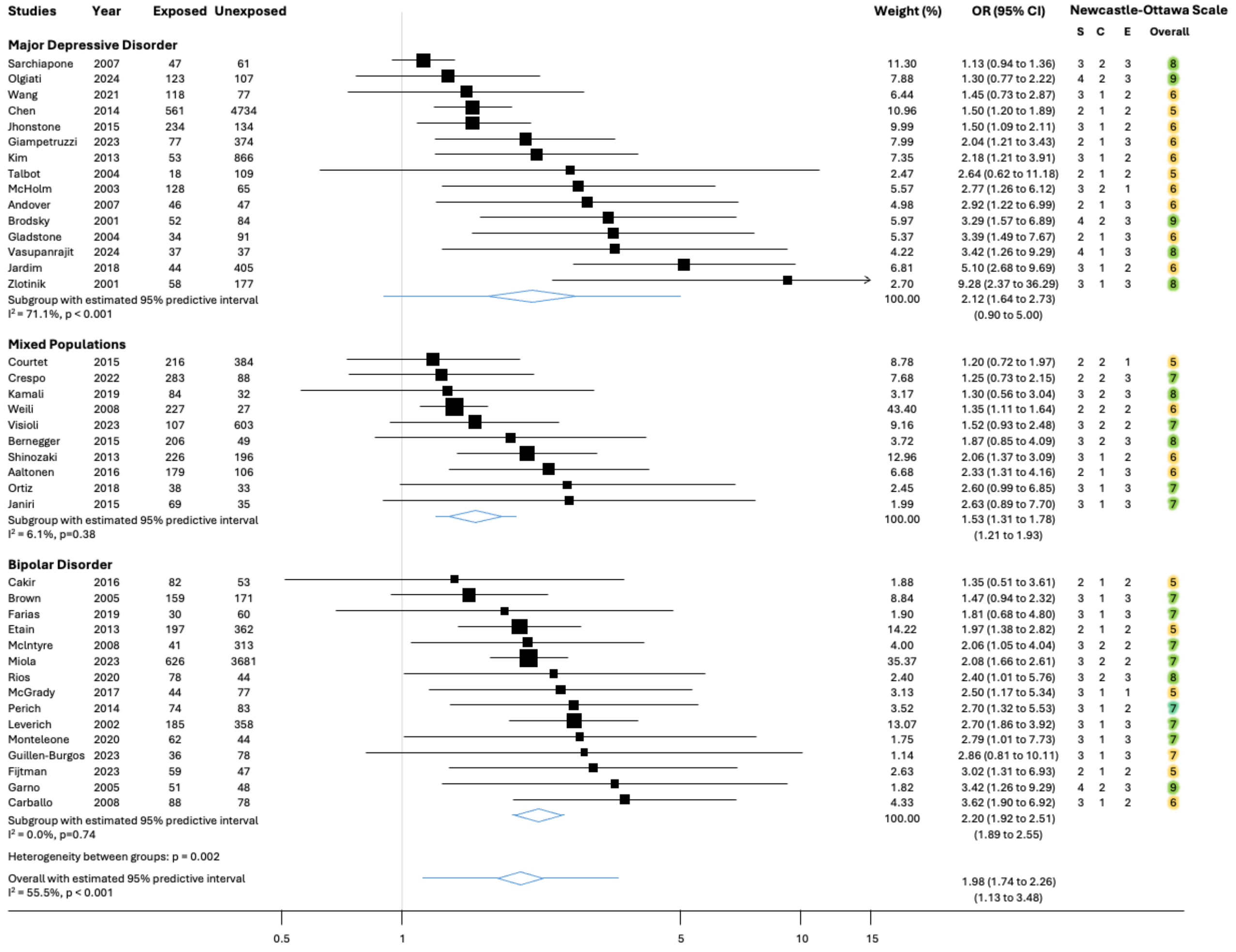

Primary outcome: Umbrella review criteria

Classification	Criteria
Convincing evidence (Class I)	✓ More than 1000 cases<br>✓ Significant summary associations ($p<10^{-6}$) per random-effects calculations<br>✓ No evidence of small-study effects<br>✓ No evidence of excess of significance bias<br>✓ Prediction intervals not including the null value<br>✓ Largest study nominally significant ($p<0.05$)<br>✓ Not large heterogeneity (i.e., $I^2 < 50\%$)
Highly Suggestive evidence (Class II)	✓ More than 1000 cases<br>✓ Significant summary associations ($p<10^{-6}$) per random-effects calculation<br>✓ Largest study nominally significant ($p<0.05$)
Suggestive Evidence (Class III)	✓ More than 1000 cases<br>✓ Significant summary associations ($p<10^{-3}$) per random-effects calculations
Weak evidence	✓ All other associations with $p < 0.05$
Non-significant associations	✓ All associations with $p > 0.05$

From Machado et al. BMC Med. 2018 Jul 20;16(1):112

Umbrella review criteria assessment:

(1) Significant summary associations ($p<10^{-6}$) per random-effects calculations

p=3.64407913092e-25 (random effects meta-analysis)

(2) Small-study effects

(a) Egger's regression asymmetry test (P≤0.10)

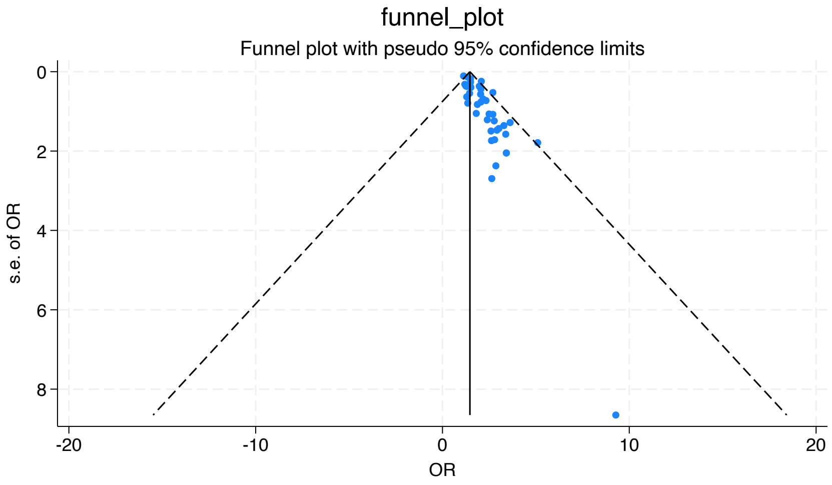

```
Number of studies =  40                                 Root MSE      =   .7798
------------------------------------------------------------------------------
     Std_Eff | Coefficient  Std. err.      t    P>|t|     [95% conf. interval]
-------------+----------------------------------------------------------------
       slope |   1.149902   .0645189    17.82   0.000      1.01929    1.280513
        bias |   1.218567   .1730763     7.04   0.000      .868192    1.568941
------------------------------------------------------------------------------

Test of H0: no small-study effects          P = 0.000
```

(b) whether the random effects summary estimate is larger than the point estimate of the largest study in each association.

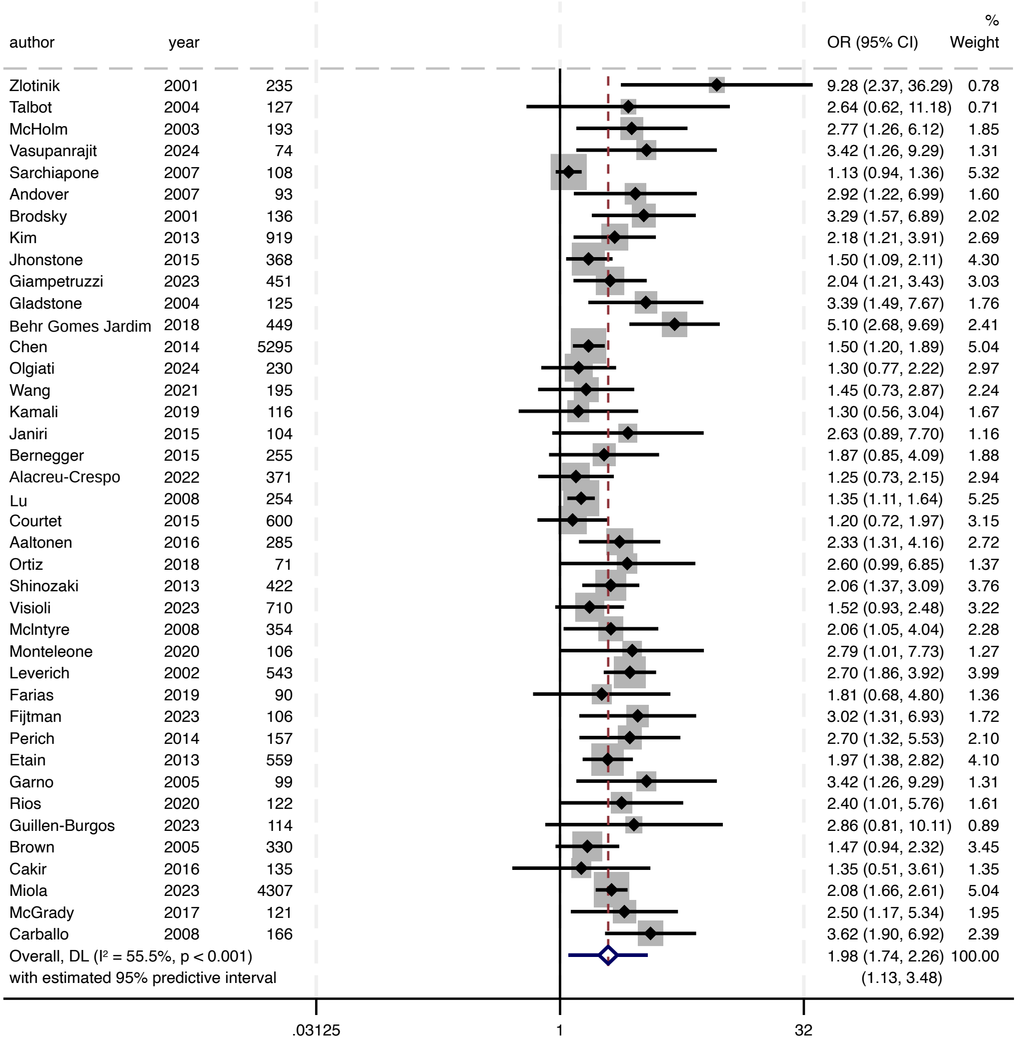

Interpretation: there is a high risk of small-study effect bias

(3) Excess of significance Bias

signBiasTester power lnrr rrse

The command provides a table reporting expected and observed studies. The Excess of significance Bias occurs when the p-value (pBin) is ≤0.10.

```
Disagreements in the number of observed and expected significant studies

  +-------------------------------------------------------------------+
  | M-A    N   Expected   Observed   pChi   pBin   pBin, more   pBin, less |
  |-------------------------------------------------------------------|
  |   1   40      27.29          3   0.00   0.00         1.00         0.00 |
  +-------------------------------------------------------------------+
```

Interpretation: there is a high risk of excess of significance bias

(4) Prediction intervals not including the null value

Interpretation: predictive intervals do not include the null value (see forest plot above)

Overall interpretation:

	#1		#2	#3			#4	#5	#6	#7	Class
**Outcome**	**Number of cases**	**More than 1000?**	**p-value ES random effect MA ($<10^{-6}$?)**	**Egger's regression asymmetry test (p≤0.10)**	**RE MA estimate larger of the largest study**	**No small study effect**	**No excess of significance bias (p≤0.10)**	**Predictive intervals not including the null value**	**Largest study nominally significant (p<0.05)**	**Not large heterogeneity (< 50%)**	
Any ACE, any suicide behavior	3888	YES	YES	YES	YES	NO	NO	YES	YES	NO	Class II - highly suggestive evidence

Secondary analyses

Sensitivity analyses and/or meta-regression analyses on having children, level of education, employment, being religious, being physically ill, family history of suicide behaviors, duration of mental illness, number of maniac/depressive episodes, number of previous hospitalizations for mental illness, comorbid PTSD, comorbid personality disorders, comorbid psychotic features, rapid cycling, aggressivity, and issues with justice were not performed as data reported in original studies were too few.

We were not able to analyze the risk of suicide behavior as a function of the severity of ACEs as data reported in original studies were too few.

Secondary outcomes “death by suicide” and “non-suicidal self-injury” in people exposed to any ACE were not performed as data reported in original studies were too few.

We were not able to meta-analyze the risk of suicide behavior as a function of the CTQ-SF score, as few studies provided this data.

Sensitivity analysis: removing studies for which data were imputed from CTQ mean scores

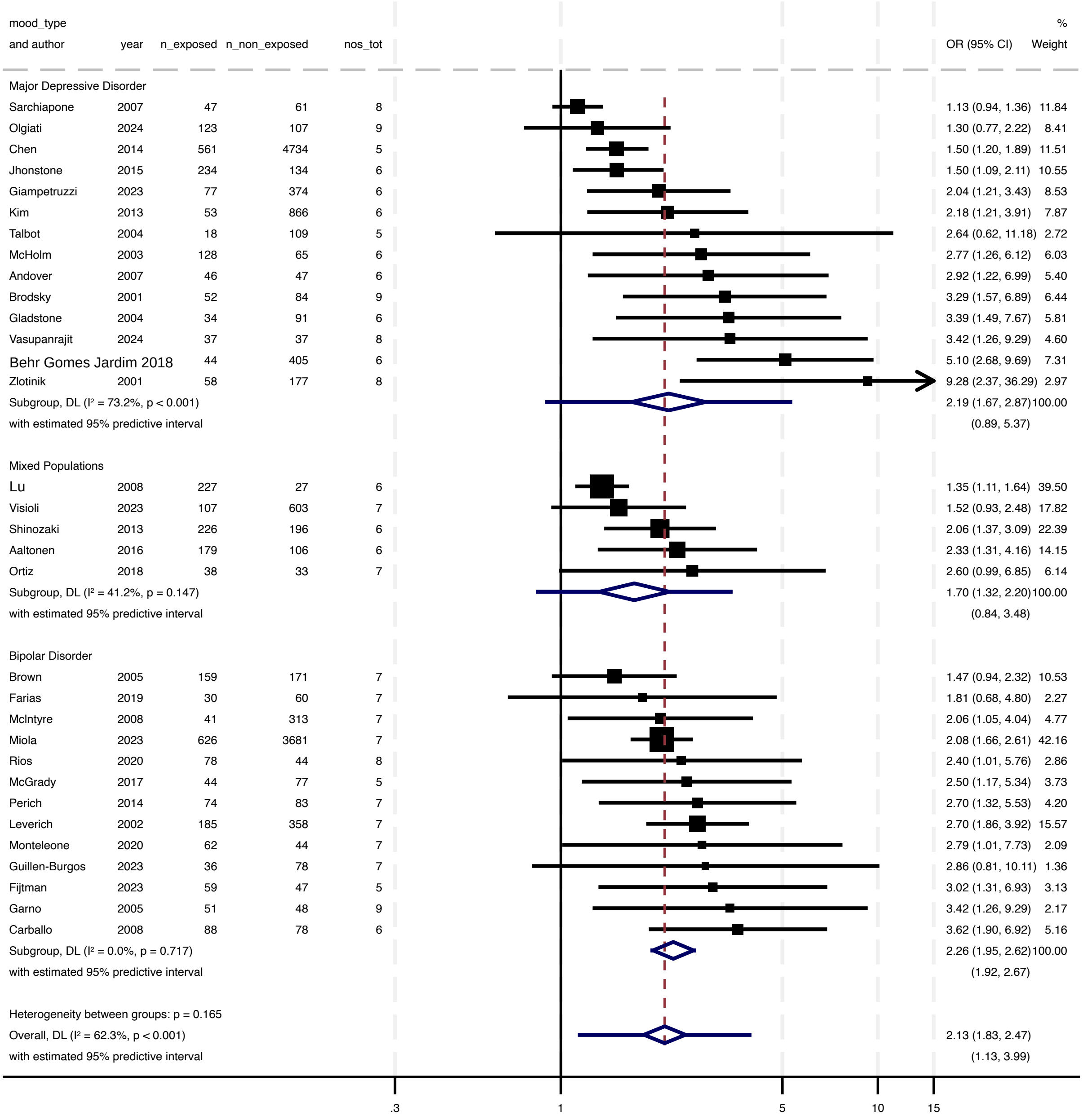

NOTE: Weights and between-subgroup heterogeneity test are from random-effects model

Sensitivity analysis: removing studies for which adjusted OR was not available

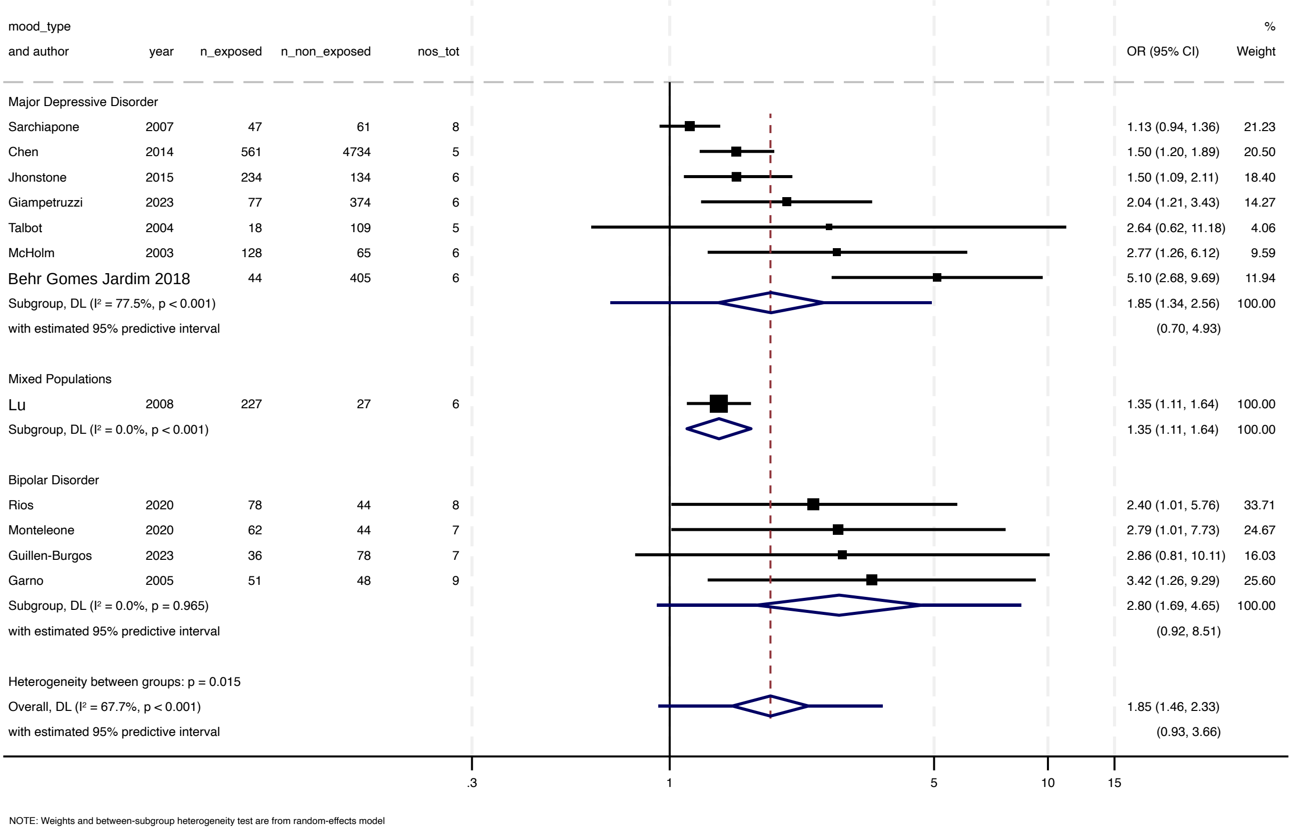

Sensitivity analysis: removing studies with NOS score ≤5

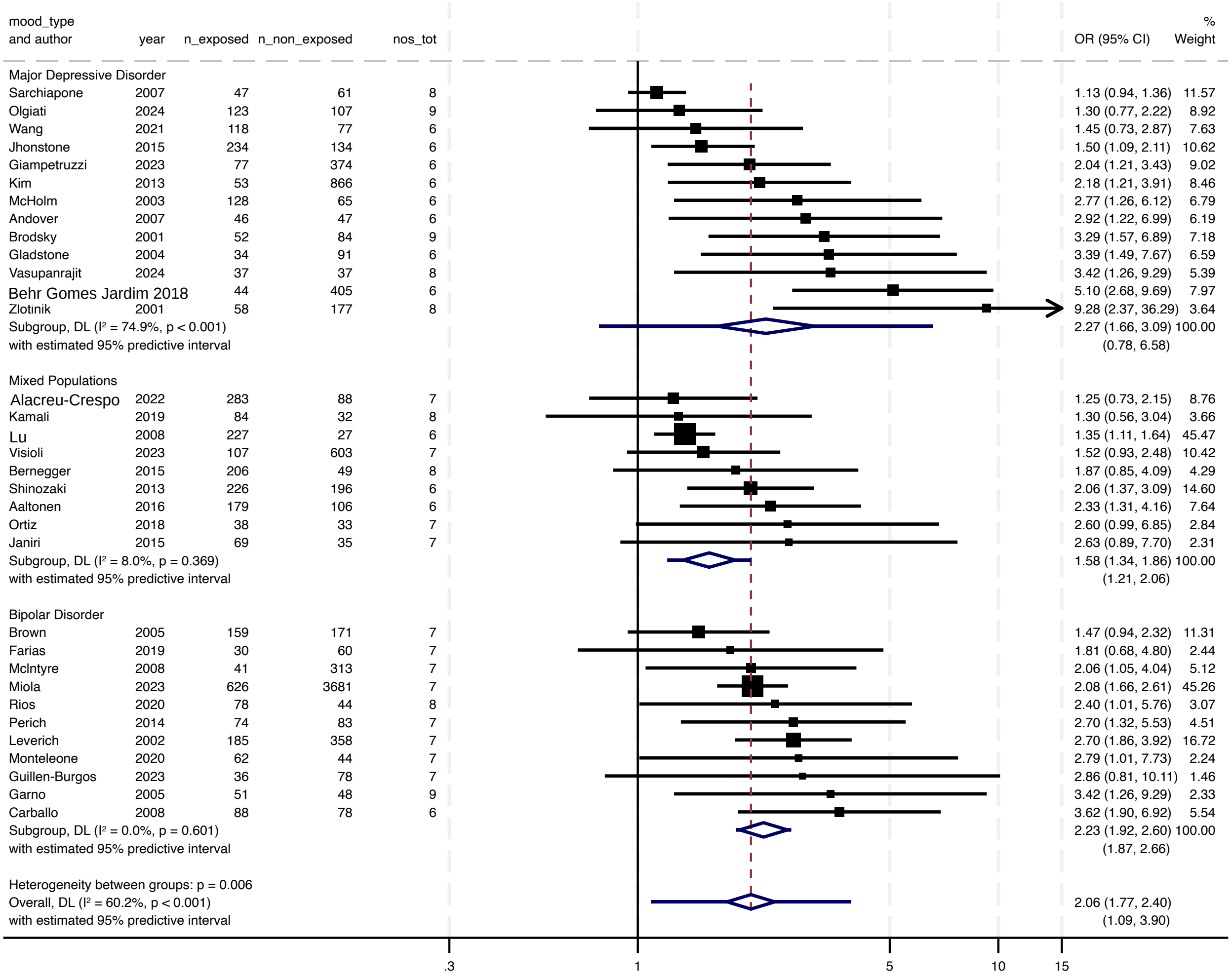

NOTE: Weights and between-subgroup heterogeneity test are from random-effects model

Meta-regression analysis: percentage of females

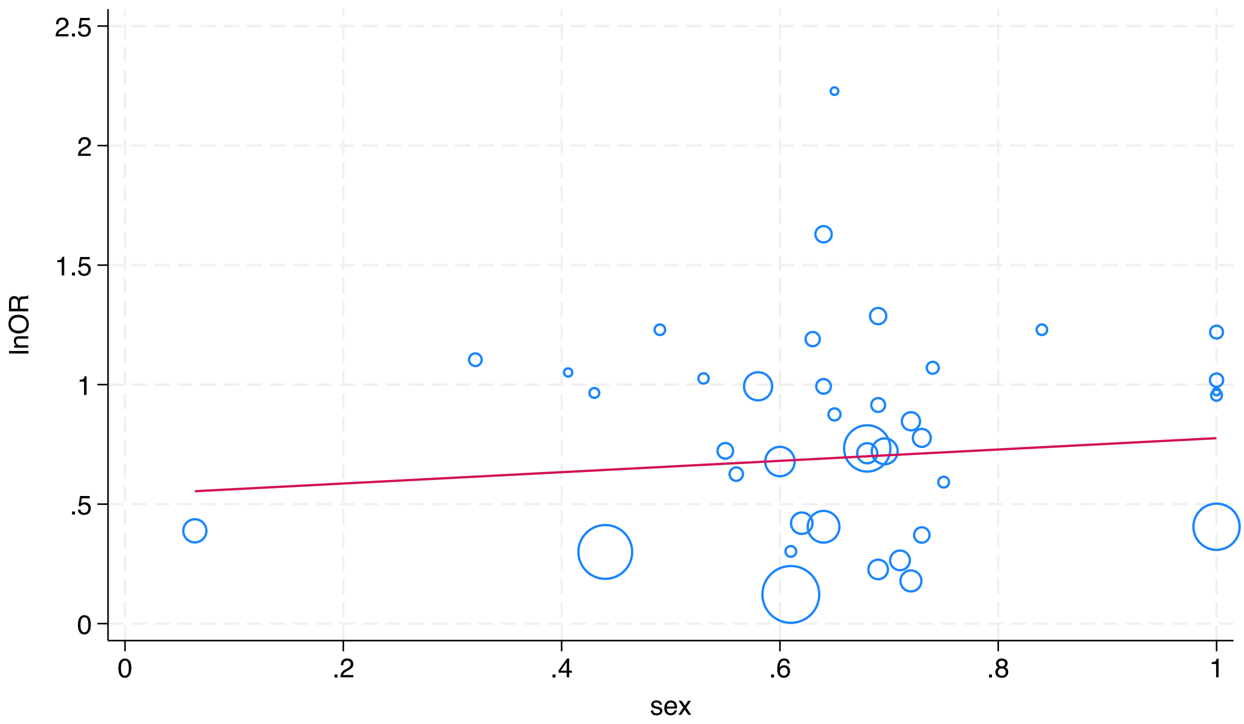


```
Meta-regression                                     Number of obs  =        39
REML estimate of between-study variance             tau2           =    .07556
% residual variation due to heterogeneity           I-squared_res  =    56.85%
Proportion of between-study variance explained      Adj R-squared  =    -3.53%
With Knapp-Hartung modification
------------------------------------------------------------------------------
        lnOR | Coefficient  Std. err.      t    P>|t|     [95% conf. interval]
-------------+----------------------------------------------------------------
         sex |   .2373127   .3649594     0.65   0.520    -.5021653    .9767907
       _cons |   .5387172   .2468643     2.18   0.036     .0385225    1.038912
------------------------------------------------------------------------------
```

Meta-regression analysis: mean age

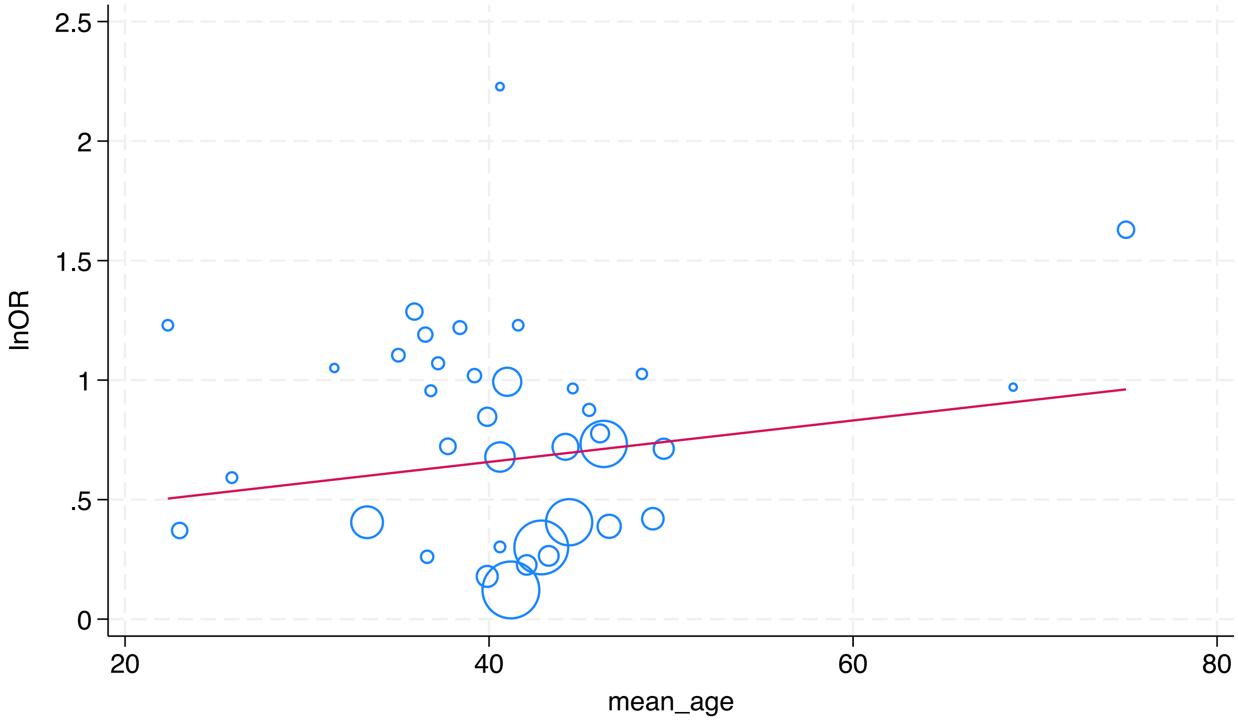


```
Meta-regression                                    Number of obs  =       37
REML estimate of between-study variance            tau2           =   .07334
% residual variation due to heterogeneity          I-squared_res  =   57.19%
Proportion of between-study variance explained     Adj R-squared  =    2.53%
With Knapp-Hartung modification
------------------------------------------------------------------------------
        lnOR | Coefficient  Std. err.      t    P>|t|     [95% conf. interval]
-------------+----------------------------------------------------------------
    mean_age |    .008678    .008267     1.05   0.301    -.0081048    .0254609
       _cons |   .3105181   .3528661     0.88   0.385    -.4058381    1.026874
------------------------------------------------------------------------------
```

Meta-regression analysis: percentage of individuals with bipolar disorder

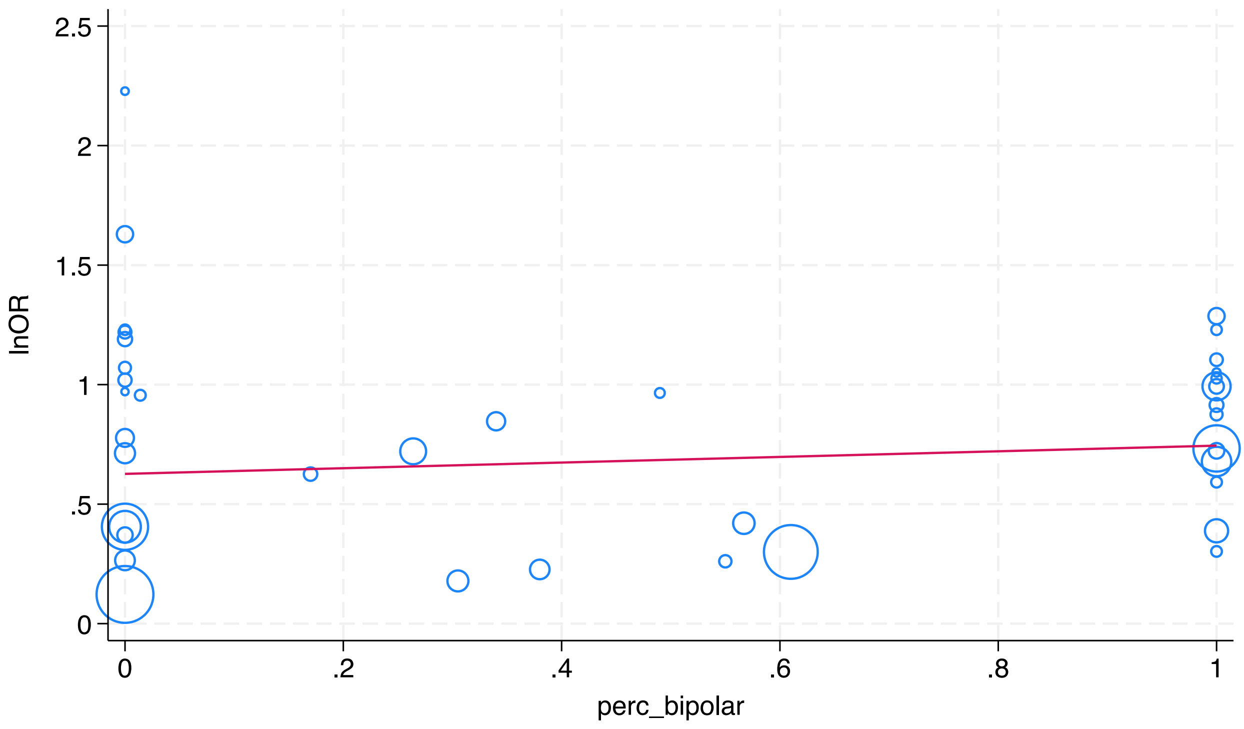


```
Meta-regression                                   Number of obs  =       40
REML estimate of between-study variance           tau2           =   .06771
% residual variation due to heterogeneity         I-squared_res  =   50.72%
Proportion of between-study variance explained    Adj R-squared  =    4.74%
With Knapp-Hartung modification
------------------------------------------------------------------------------
        lnOR | Coefficient  Std. err.      t    P>|t|     [95% conf. interval]
-------------+----------------------------------------------------------------
perc_bipolar |   .1181213   .1482465     0.80   0.431    -.1819882    .4182307
       _cons |   .6266411   .0936369     6.69   0.000     .4370832    .8161991
------------------------------------------------------------------------------
```

Meta-regression analysis: percentage of married participants

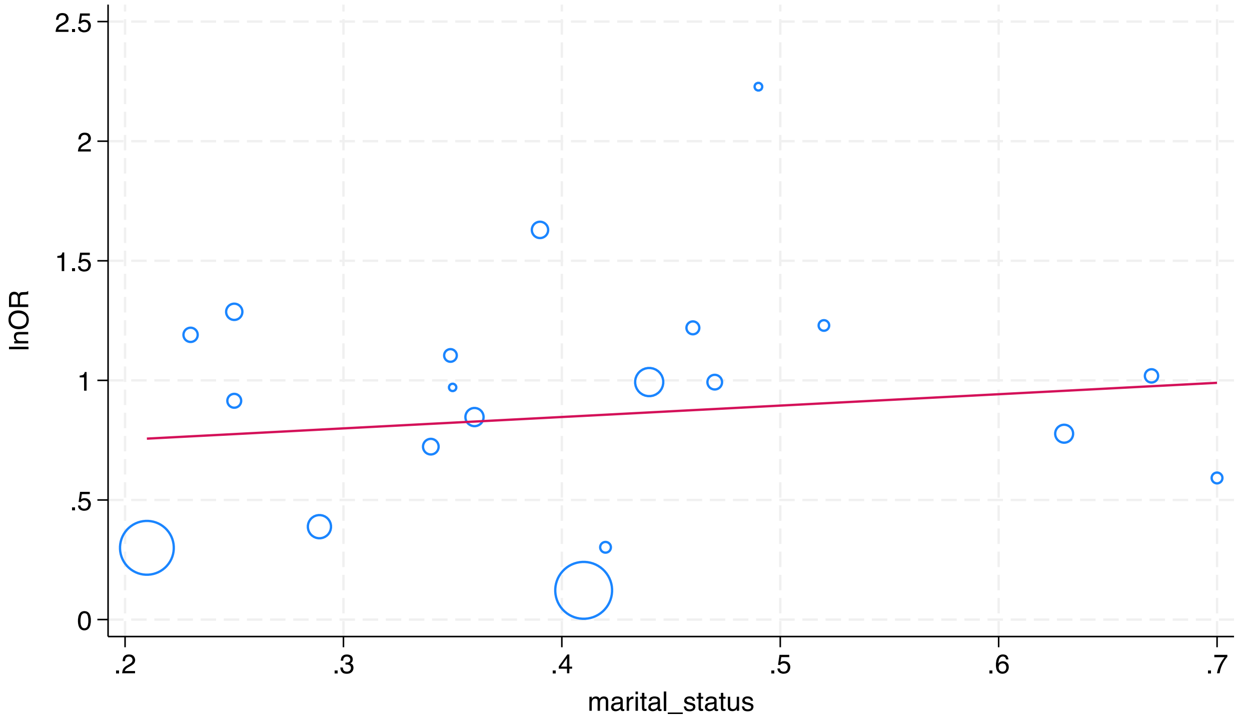


```
Meta-regression                                   Number of obs  =       20
REML estimate of between-study variance           tau2           =    .1371
% residual variation due to heterogeneity         I-squared_res  =   72.51%
Proportion of between-study variance explained    Adj R-squared  =   -2.13%
With Knapp-Hartung modification
--------------------------------------------------------------------------------
          lnOR | Coefficient  Std. err.      t    P>|t|     [95% conf. interval]
---------------+----------------------------------------------------------------
marital_status |   .4762659   .8404079     0.57   0.578    -1.289366    2.241897
         _cons |   .6564557   .3512939     1.87   0.078    -.0815854    1.394497
--------------------------------------------------------------------------------
```

Meta-regression analysis: percentage of participants with current alcohol/substance misuse

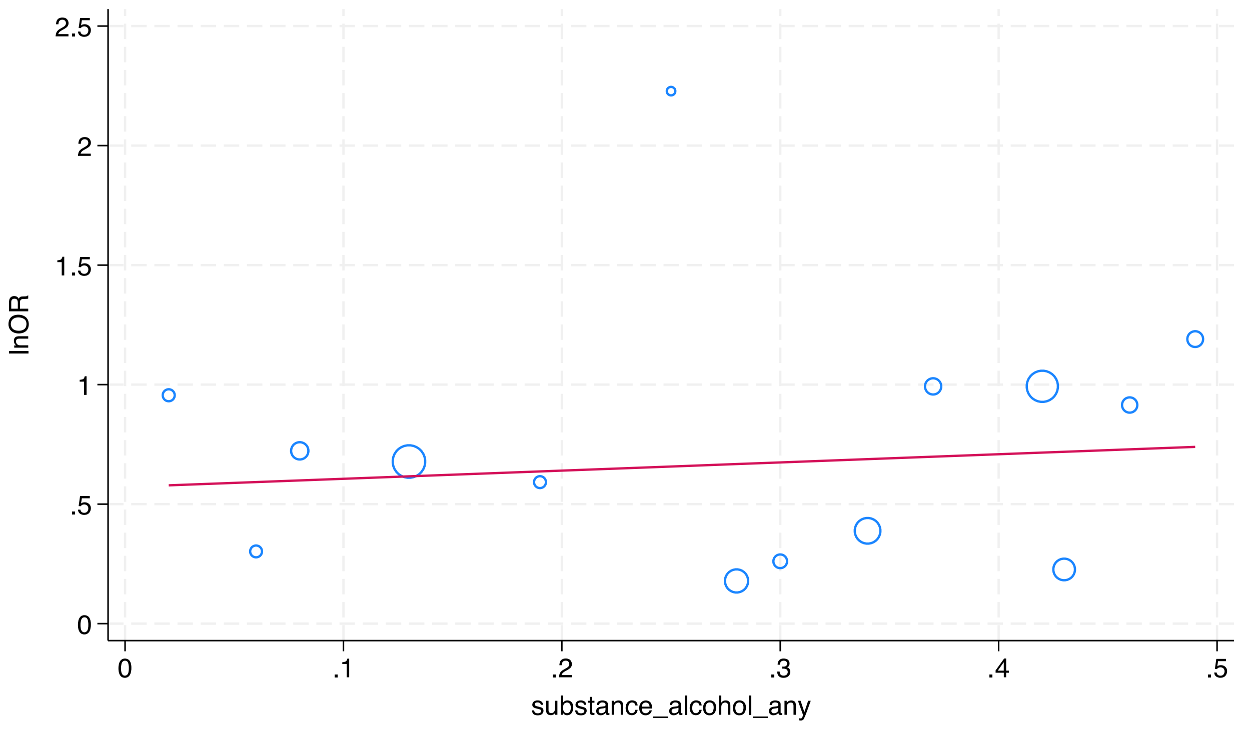


```
Meta-regression                                     Number of obs  =      14
REML estimate of between-study variance             tau2           =  .05644
% residual variation due to heterogeneity           I-squared_res  =  40.46%
Proportion of between-study variance explained      Adj R-squared  = -19.87%
With Knapp-Hartung modification
--------------------------------------------------------------------------------------
                 lnOR | Coefficient  Std. err.      t    P>|t|     [95% conf. interval]
---------------------+----------------------------------------------------------------
substance_alcohol_any |   .3423131   .8013522     0.43   0.677    -1.403683     2.08831
                _cons |   .5718092   .2600369     2.20   0.048     .0052374    1.138381
--------------------------------------------------------------------------------------
```

Meta-regression analysis: percentage of participants with any comorbid mental health condition

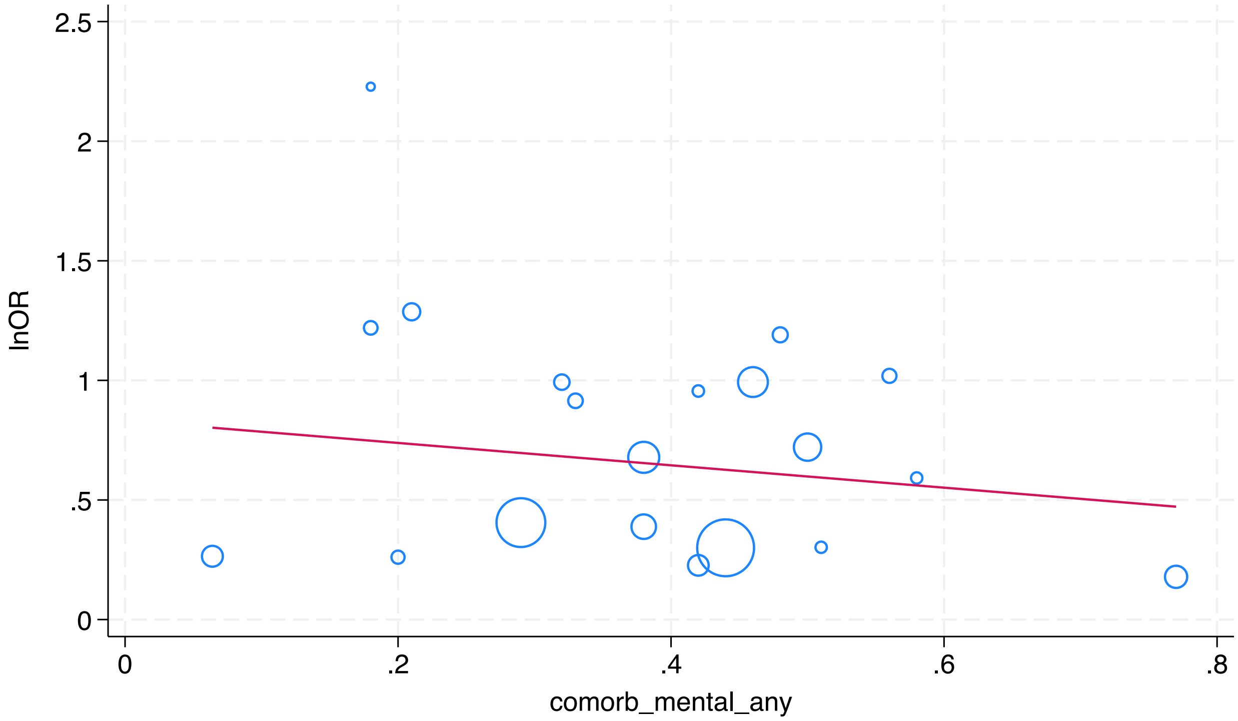


```
Meta-regression                                         Number of obs  =      20
REML estimate of between-study variance                 tau2           =  .07403
% residual variation due to heterogeneity               I-squared_res  =  55.24%
Proportion of between-study variance explained          Adj R-squared  = -11.71%
With Knapp-Hartung modification
------------------------------------------------------------------------------------
            lnOR | Coefficient  Std. err.      t    P>|t|     [95% conf. interval]
-----------------+------------------------------------------------------------------
comorb_mental_any |  -.4676122   .5943517    -0.79   0.442    -1.716299    .7810744
           _cons |   .8320408   .2509066     3.32   0.004     .3049057    1.359176
------------------------------------------------------------------------------------
```

Meta-regression analysis: percentage of participants with comorbid anxiety

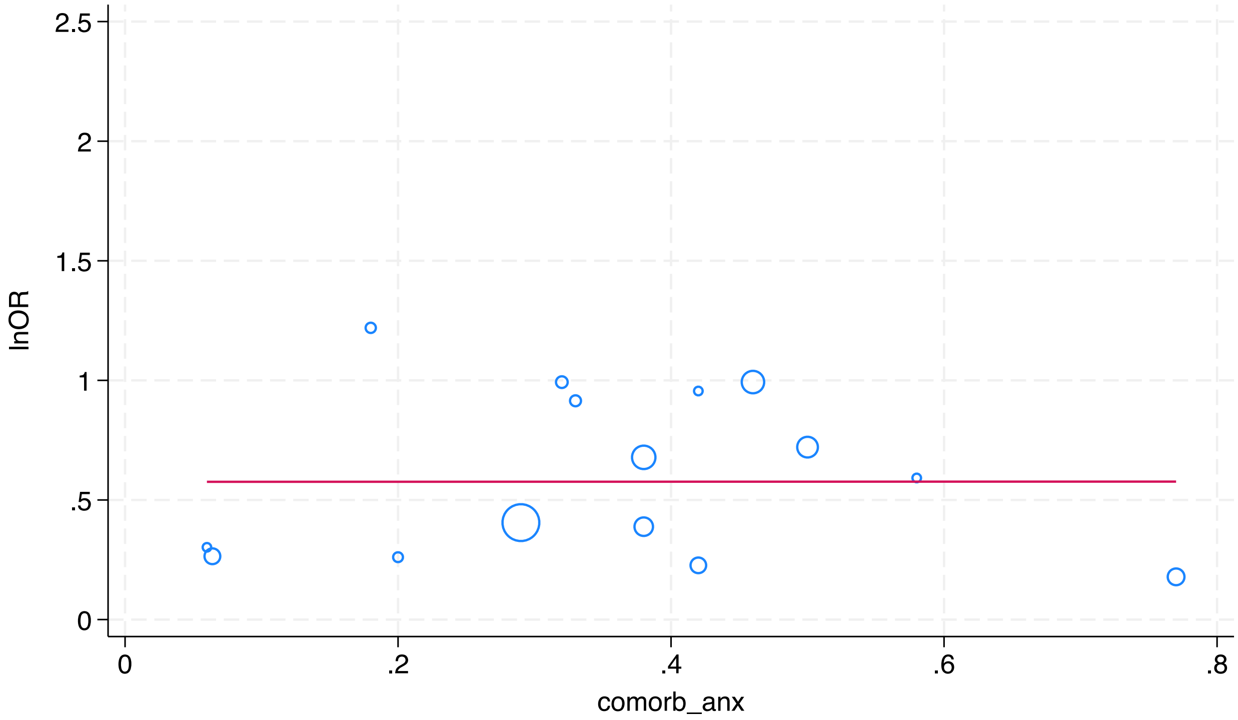


```
Meta-regression                                    Number of obs  =        15
REML estimate of between-study variance            tau2           =    .03677
% residual variation due to heterogeneity          I-squared_res  =    32.99%
Proportion of between-study variance explained     Adj R-squared  =   -21.62%
With Knapp-Hartung modification
------------------------------------------------------------------------------
        lnOR | Coefficient  Std. err.      t    P>|t|     [95% conf. interval]
-------------+----------------------------------------------------------------
  comorb_anx |   .0011803   .5193825     0.00   0.998    -1.120877    1.123238
       _cons |   .5759288   .2140995     2.69   0.019      .113395    1.038463
------------------------------------------------------------------------------
```

Secondary analysis: sexual abuse, any suicidal behavior

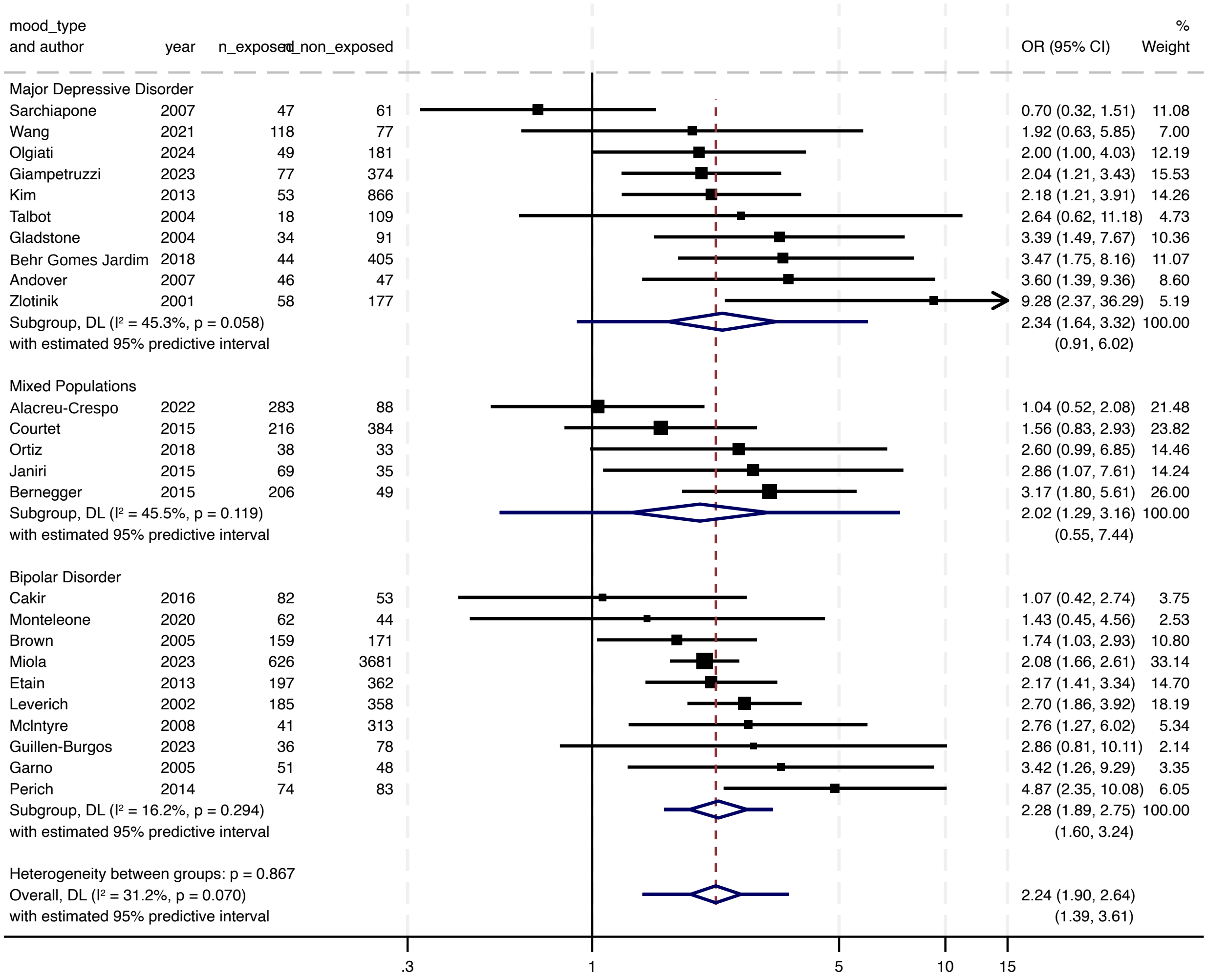

NOTE: Weights and between-subgroup heterogeneity test are from random-effects model

Secondary analysis: physical abuse, any suicidal behavior

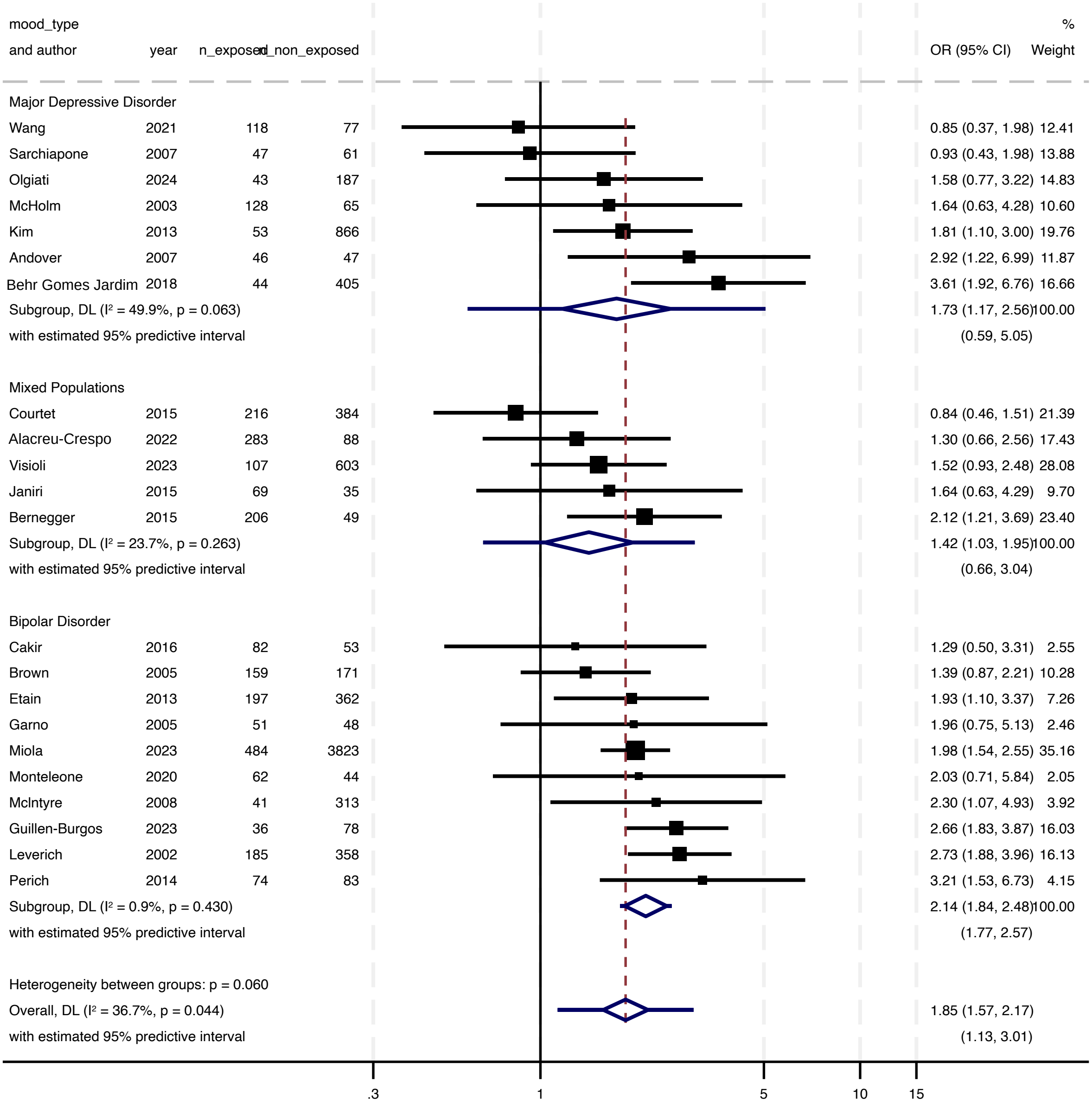

NOTE: Weights and between-subgroup heterogeneity test are from random-effects model

Secondary analysis: physical neglect, any suicidal behavior

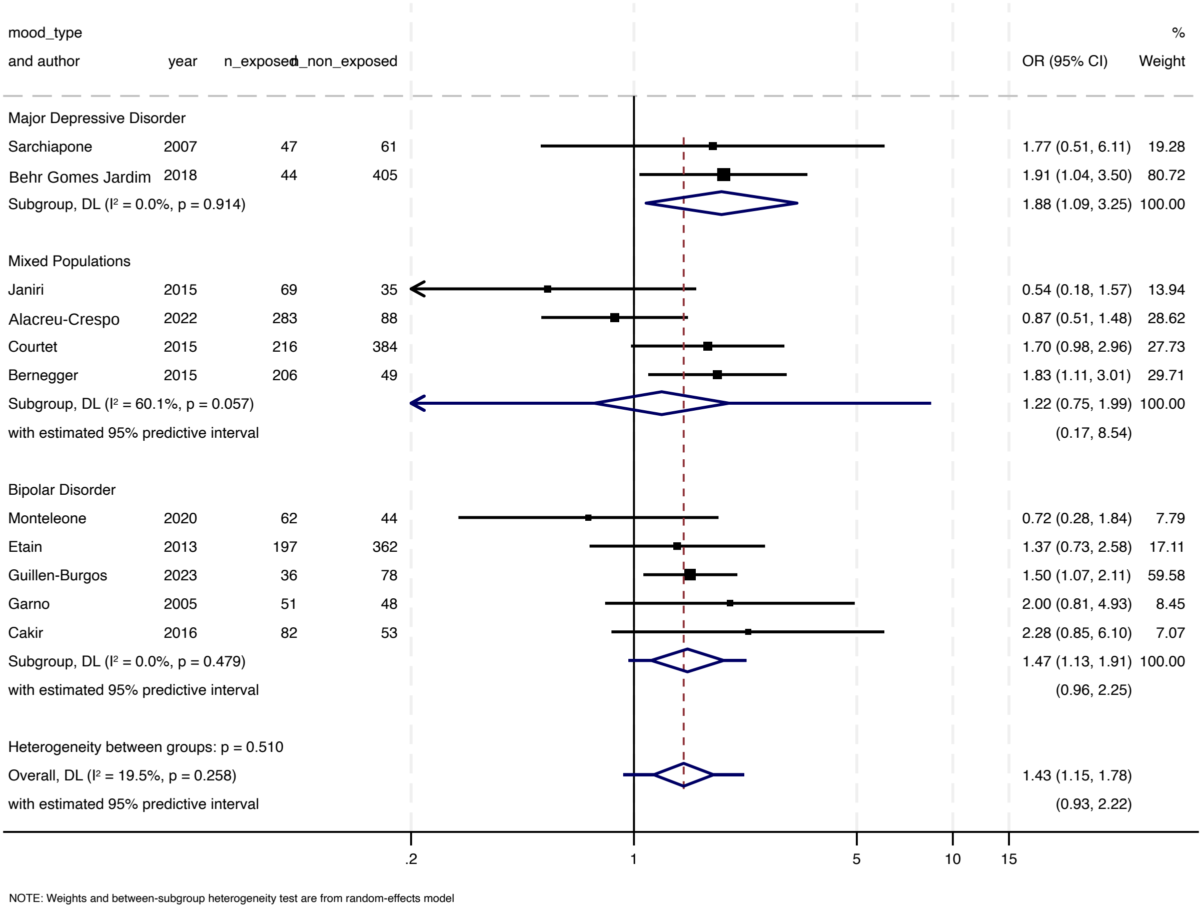

Secondary analysis: emotional abuse, any suicidal behavior

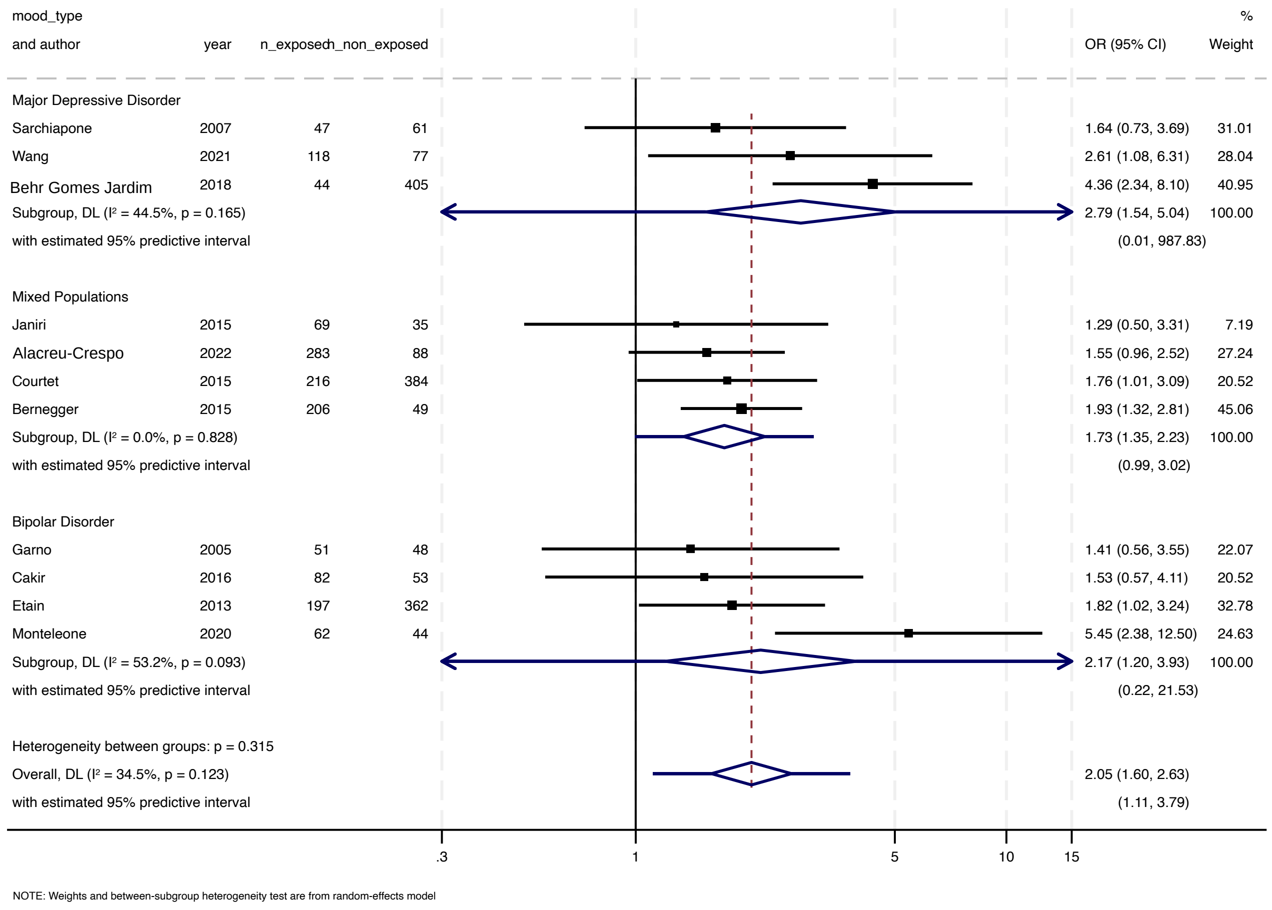

Secondary analysis: emotional neglect, any suicidal behavior

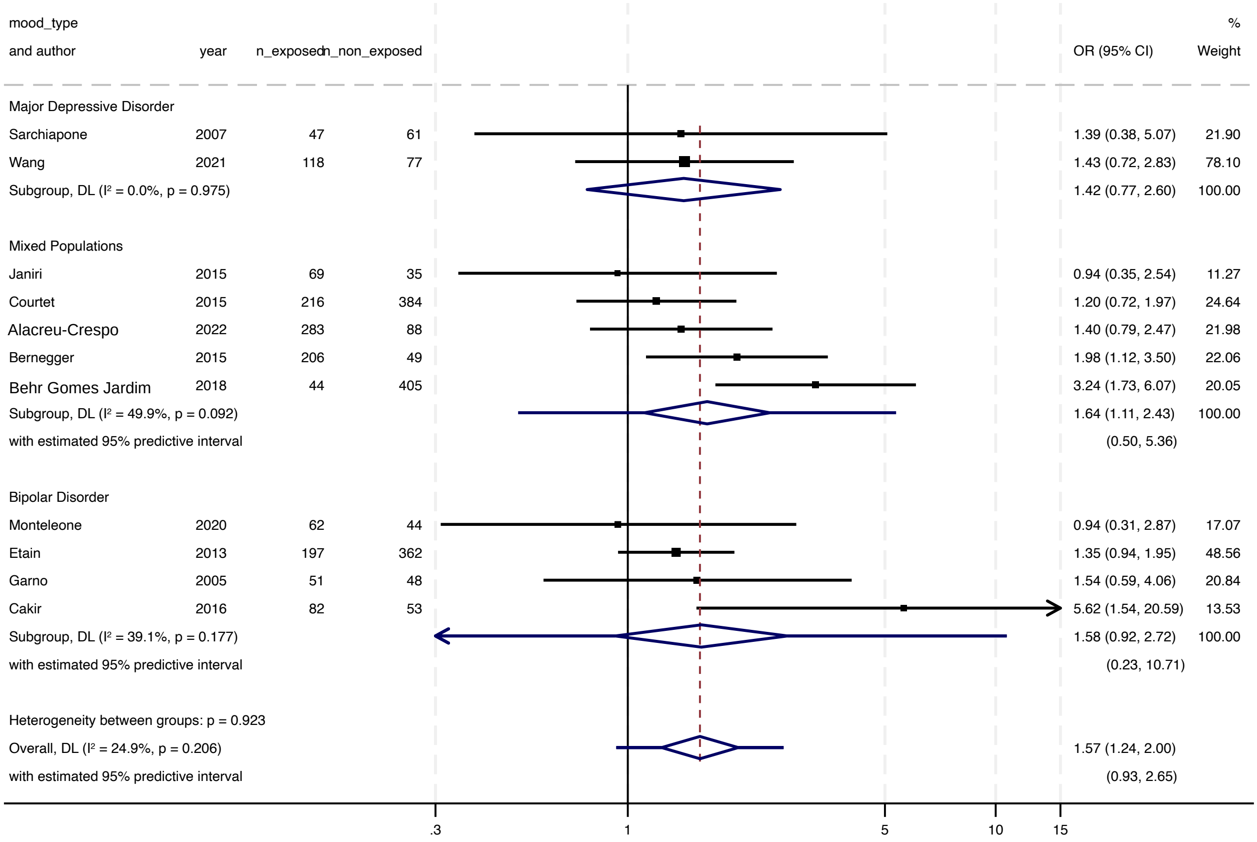

NOTE: Weights and between-subgroup heterogeneity test are from random-effects model

Secondary analysis: any ACE, suicide ideation

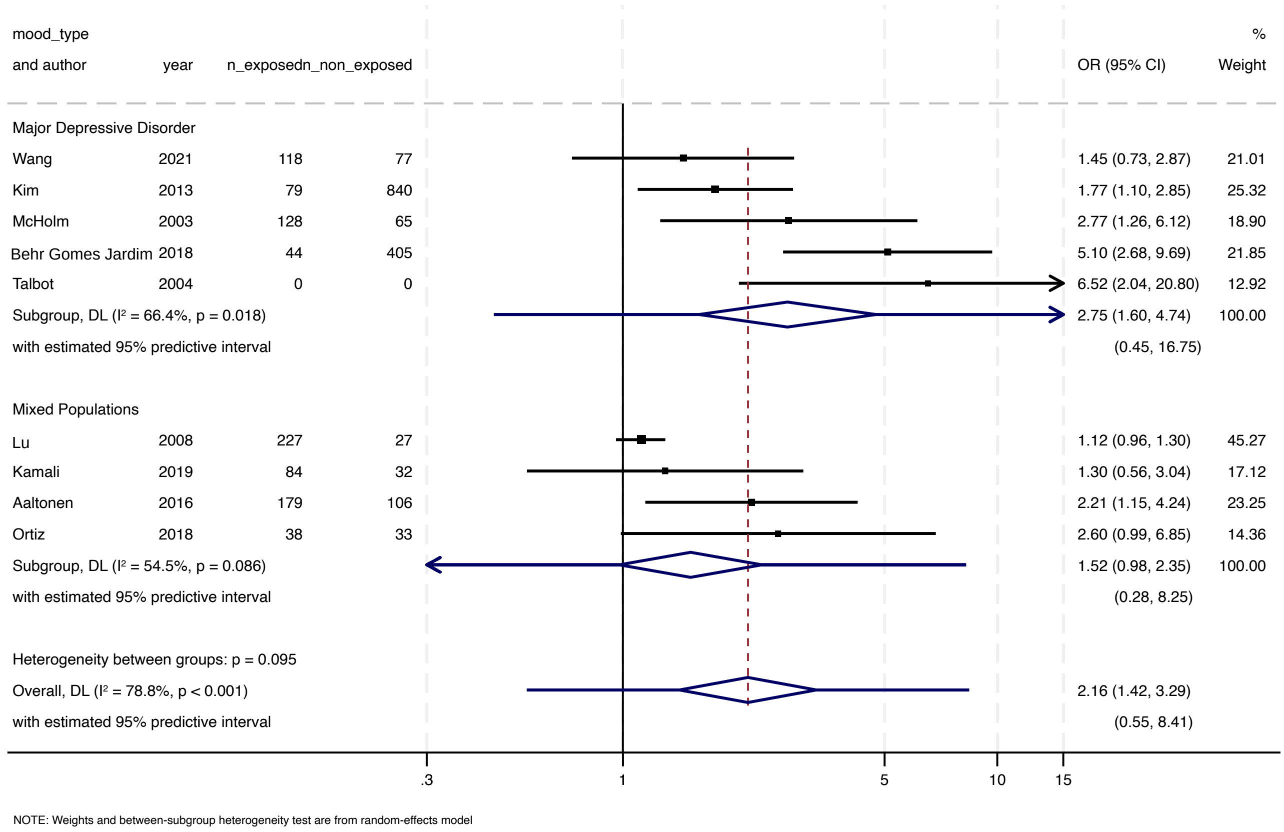

Secondary analysis: any ACE, suicide attempt

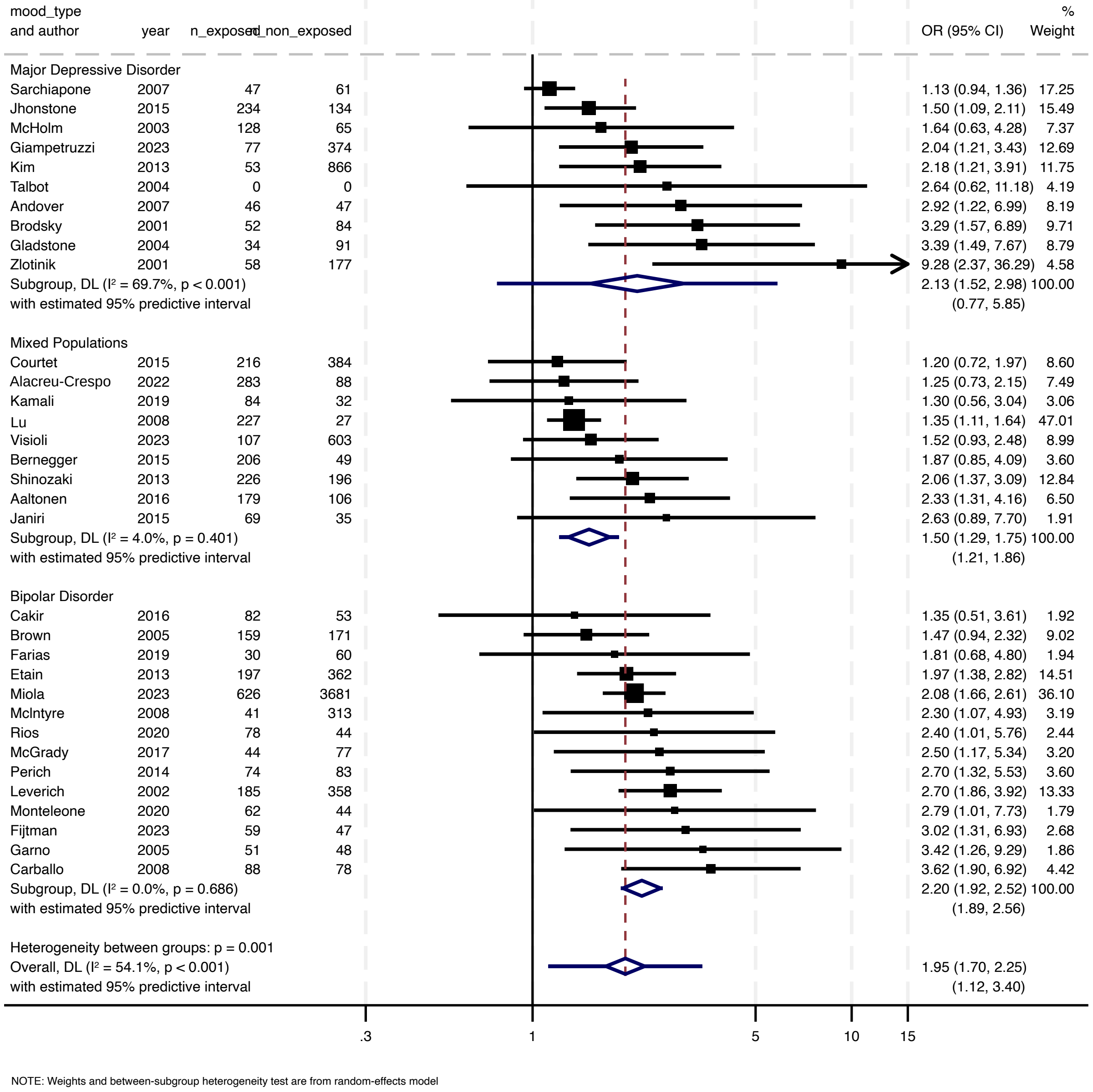

Supplement: Baldini et al. supplementary material [file S0924933825024526sup001.pdf]
